# Reprogrammed glucose metabolism in vascular smooth muscle cells and its implications for vascular diseases

**DOI:** 10.1016/j.phrs.2025.107995

**Published:** 2025-10-13

**Authors:** Qian Ma, Yongfeng Cai, Qiuhua Yang, Wendy Zhang, Suowen Xu, Yuqing Huo

**Affiliations:** a Departments of Ophthalmology, Medicine, and Molecular and Cellular Biology, Baylor College of Medicine, Houston, TX 77030, USA; b Department of Pharmacological Sciences, Stony Brook University, Stony Brook, NY 11794, USA; c Rowan-Virtua School of Osteopathic Medicine, Stratford, NJ 08084, USA; d Institute of Endocrine and Metabolic Diseases, the First Affiliated Hospital of USTC, Division of Life Sciences and Medicine, University of Science and Technology of China, Hefei, Anhui 230001, China

**Keywords:** Vascular Smooth Muscle cell, Glucose Metabolism, Atherosclerosis, Calcification, Aneurysm, Pulmonary Hypertension

## Abstract

Vascular smooth muscle cells (VSMCs) play a pivotal role in maintaining vascular homeostasis and are critical contributors to the pathogenesis of various vascular diseases, including atherosclerosis, calcification, aneurysms, and pulmonary hypertension. Emerging evidence highlights the significance of glucose metabolism in regulating VSMC phenotypic transitions during these pathologies. This review provides a comprehensive overview of the major glucose metabolic pathways in VSMCs, glycolysis, the pentose phosphate pathway (PPP), and the hexosamine biosynthetic pathway (HBP), and their roles in disease development. We summarize the molecular mechanisms linking glucose metabolic reprogramming to VSMC dysfunction, focusing on key regulatory enzymes and signaling pathways. Additionally, we discuss the translational potential of targeting glucose metabolism in VSMCs for therapeutic intervention and the challenges associated with this approach. By underscoring the metabolic shifts underlying VSMC pathophysiology, this review aims to advocate further research into VSMC glucose metabolism as a promising avenue for novel vascular disease treatments.

## 1. Introduction

Vascular smooth muscle cells (VSMCs) are the predominant cell type within the medial layers of arteries. In healthy arteries, VSMCs express contractile proteins including α-smooth muscle actin (αSMA) and smooth muscle myosin heavy chain (SMMHC; also known as myosin 11 (MYH11), and exhibit a contractile phenotype that contributes to the regulation of blood vessel tone, blood flow distribution, and blood pressure in normal mature blood vessels [1]. VSMCs will conduct phenotypic transition characterized by the expression of markers of alternative cell types (eg, macrophage-like, mesenchymal-stem cell-like, osteoblast-like) under pathological conditions or stimulators [2–4]. This transition is critical for vascular disease development, such as atherosclerosis [5–7], aneurysm [8,9], calcification [9,10], pulmonary hypertension [11,12], etc. Thus, targeting VSMC phenotypic transition is critical in the treatment of vascular diseases.

Over the past few decades, our understanding of cellular metabolism regulation has advanced significantly, particularly regarding the interplay between cellular metabolism, disease risk factors, and signaling pathways. Increasing evidence shows a close association between vascular diseases and metabolism in vascular cells, including VSMCs [13], endothelial cells (EC) [14] and macrophages [15]. Among all aberrant changes, alterations in the metabolism and bioenergetics of VSMCs are increasingly recognized as universal hallmarks of the pathobiology of vascular diseases. Targeting the altered metabolism in VSMCs, including fatty acid metabolism [16], amino acid metabolism [17], nucleotide metabolism [18,19], and glucose metabolism [20], could be a promising strategy for the treatment of vascular diseases. Studies indicate that glucose metabolic reprogramming may be involved in the phenotypic transition of VSMCs in vascular diseases. In the present review, we reviewed the current publications about the significant glucose metabolic pathways, glycolysis, pentose phosphate pathway (PPP), and hexosamine biosynthesis pathway (HBP), of VSMCs in vascular diseases. This review will provide information on how glucose metabolism is regulated in VSMCs, how it influences VSMC pathogenic phenotypes, and whether these pathways can be targeted for therapeutic interventions in vascular diseases (Table 1).

## 2. Glucose metabolism

**Fig. 1.**
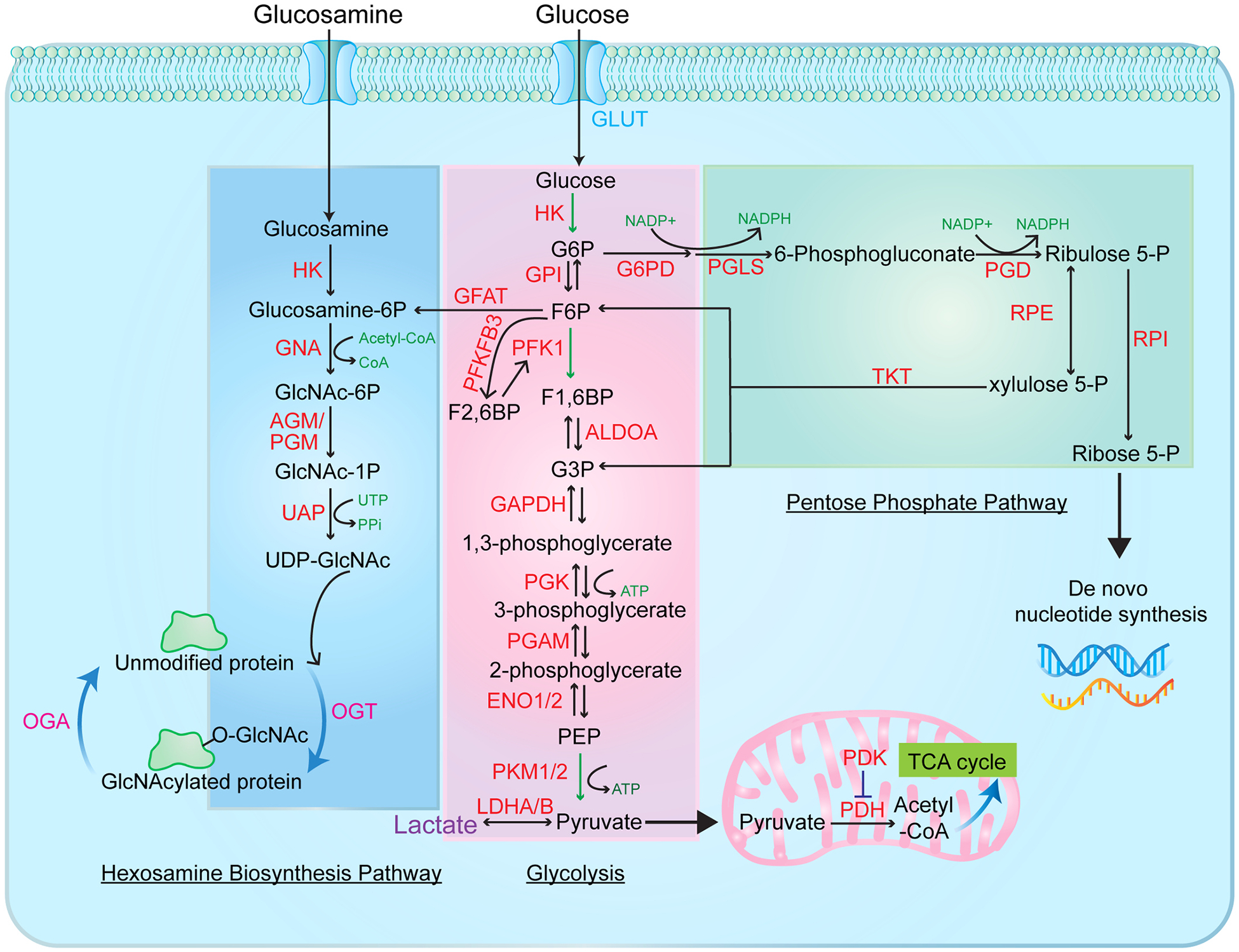
The glucose metabolism in vascular smooth muscle cells (VSMCs). Schematic overview of the glucose metabolic pathways and their known metabolic enzymes in VSMCs. GLUT, glucose transporter; HK, hexokinase; GPI, glucose-6-phosphate isomerase; G6P, glucose-6-phosphate; PFK1, 6-phosphofructo-1 kinase; F6P, fructose-6-phosphate; F1,6BP, fructose-2,6-bisphosphate; F2,6BP, fructose-2,6-bisphosphate; PFKFB3, 6-phosphofructo-2-kinase/fructose-2,6-bisphosphatase isoform 3; ALDOA, aldolase A; PGAM, phosphoglycerate mutase; ENO1/2, enolase 1/2; GAPDH, glyceraldehyde-3-phosphate dehydrogenase; PGK, phosphoglycerate kinase; LDHA/B, lactate dehydrogenase subunit A/B; PEP, phosphoenolpyruvate; PKM1/2, pyruvate kinase muscle isozyme 1/2; PDK, pyruvate dehydrogenase kinase; PDH, pyruvate dehydrogenase; TCA, tricarboxylic acid; NADPH, nicotinamide adenine dinucleotide phosphate; GFAT, glutamine fructose-6-phosphate aminotransferase; GNA, glucosamine 6-phosphate N-acetyltransferase; AGM/PGM, phosphoglucomutase; UAP, UDP-N-acetylhexosamine pyrophosphorylase; UTP, uridine triphosphate; OGA, O-GlcNAcase; OGT, O-GlcNAc transferase; UDP-GlcNAc, uridine diphosphate N-acetylglucosamine; O-GlcNAc, O-linked N-acetylglucosamine; G6PD, glucose-6-phosphate dehydrogenase; PGLS, 6-phosphogluconolactonase; PGD, 6-phosphogluconate dehydrogenase; RPE, ribulose 5-phosphate epimerase; RPI, ribose 5-phosphate isomerase; TKT, transketolase.

Cellular metabolism is the sum of all chemical changes that take place in a cell through which energy and basic components are provided for essential processes, including the synthesis and breakdown of fatty acids, amino acids, nucleic acids, and carbohydrates. Therefore, cellular metabolism is required for cellular function, growth, and reproduction. Under normal conditions, different metabolic pathways are fine-tuned to maintain cellular homeostasis in response to changes in the environment [21]. Glucose is central to energy consumption and serves as the primary carbon source for cellular biosynthesis. Therefore, maintaining glucose metabolic homeostasis is crucial for normal physiological functions and cellular survival. Glucose metabolism involves multiple pathways. Glucose first enters cells through the glucose transporters (GLUTs) and is then converted into glucose-6-phosphate (G6P) by hexokinase (HK). G6P can subsequently be used by various metabolic pathways, such as glycolysis, PPP, and HBP (Fig. 1).

Glycolysis is the major glucose metabolism pathway, which converts G6P to lactate and produces ATP molecules through ten enzymes, including glucose-6-phosphate isomerase (GPI), 6-phosphofructo-1 kinase (PFK1), glyceraldehyde-3-phosphate dehydrogenase (GAPDH), pyruvate kinase, lactate dehydrogenase (LDH), *etc*. (Fig. 1). Pyruvate generated by pyruvate kinase can go into the tricarboxylic acid (TCA) cycle in mitochondria through an oxidation pathway. Alternatively, through aerobic glycolysis, pyruvate can also be converted to lactate, which can function as a posttranslational modification molecule [22]. Additionally, PPP is also a vital glucose metabolism pathway parallel to glycolysis. Through PPP, G6P can be used to generate ribose-5-phosphate, a precursor for nucleotide synthesis, as well as to produce nicotinamide adenine dinucleotide phosphate (NADPH), which is involved in regulating cellular redox status and the synthesis of lipids [23]. Moreover, HBP represents one accessory glucose metabolic branch in cells and governs the production of uridine diphosphate N-acetylglucosamine (UDP-GlcNAc) through several enzymes (Fig. 1). UDP-GlcNAc is the substrate of protein O-GlcNAcylation, the attachment of O-linked N-acetylglucosamine (O-GlcNAc) moieties to cytoplasmic, nuclear, and mitochondrial proteins [24]. O-GlcNAcylation is an important post-translational modification that regulates fundamental cellular processes in cells [25].

## 3. Glucose metabolism in VSMCs

Classic studies reported that vascular smooth muscle displays an unusual reliance on aerobic glycolysis high rate of lactate production despite adequate oxygenation [26,27]. Because glycolysis yields less ATP per glucose than mitochondrial oxidation, it contributed < 30 % of total ATP in those preparations, yet ≥ 90 % of consumed glucose could be recovered as lactate [26]. Importantly, these measurements were made in whole vessels, not purified VSMCs. Consistent with a VSMC-intrinsic tendency, a later study using isolated porcine and human SMCs found that ~90–95 % of glucose was converted to lactate under both normal and high-glucose conditions, whereas PPP and TCA contributions were small (~4 % and ~0.5 % at baseline; ~8 % and ~1.5 % with high glucose) [28]. Paul and colleagues proposed that glucose metabolism in VSMCs was functionally compartmentalized, with aerobic glycolysis specifically coupled to sodium and potassium transport processes, whereas oxidative metabolism was coupled to contractile energy requirements [27,29–31]. Similarly, the study from Lynch et al. showed that glucose was taken up for lactate production, whereas glycogen was preferentially used for oxidation [31]. These data indicate that the source of energy can come from different substrates, specifically ATP generated from glycolysis and respiration, which could provide the energy supply for various processes in VSMCs. Another explanation for the high aerobic glycolysis in VSMCs is that the lactate produced by aerobic glycolysis can enhance the reverse capacity of mitochondria. Although one study reported negligible lactate utilization as a fuel in isolated SMCs [28], its conclusion relied solely on ^14^CO_2_ generation from labeled lactate, an indirect readout that does not capture lactate uptake or partial metabolism. Thus, it remains unclear whether VSMCs can oxidize lactate under appropriate metabolic demands, especially given the well-supported hypothesis that lactate can be reconverted to pyruvate and oxidized in mitochondria under ATP-deficient or highly demanding conditions in non-VSMCs [32–34].

These conclusions remain influential but require careful interpretation. First of all, much of the early evidence derive from whole-vessel preparations, so endothelial, adventitial, or neural elements could contribute to the measured lactate production or oxygen use [26,27]. Furthermore, most studies relied on bulk O_2_ consumption, lactate output, CO_2_ production rather than modern ^1 3^C-tracing, leaving PPP/HBP routing, pyruvate fate, and local ATP microdomains unresolved. In summary, the addictive glycolysis metabolism of VSMCs remains poorly understood. Subsequent sections revisit this framework with more recent, mechanism-focused studies of the control nodes and disease settings that redirect glucose-carbon flow.

## 4. Metabolic Dysregulation and VSMC Pathophysiology

### 4.1. Glucose metabolism in atherosclerosis

The abnormal proliferation and migration of VSMCs are the key factors for the development of atherosclerotic processes, such as plaque formation and restenosis. Thus, studying the regulation of glucose metabolism in pathological VSMCs in atherosclerosis is very important.

In early 1959, the White Carneau (WC-As) pigeon was found to be atherosclerosis-susceptible, which naturally develops atherosclerosis, whereas Show Racer (SR-Ar) pigeons are naturally resistant to atherosclerosis [35,36]. In 1975, scientists further found significantly higher activity of two glycolytic enzymes (including phosphofructokinase (PFK) and aldolase) in arteries from the WC-As pigeon [37,38]. In 2012, researchers also confirmed that some glycolytic genes, such as enolase α (*ENO1*), glucose phosphate isomerase (*GPI*), and lactate dehydrogenase subunit A (*LDHA*), were exclusively expressed by WC-As aortic SMCs but not by SR-Ar aortic SMCs [39]. These studies suggest that increased glycolysis in the WC-As arteries may facilitate the development of atherosclerosis. In 2002, ^8^F-fluorodeoxyglucose (FDG) positron emission tomography (PET) examination showed elevated FDG uptake in symptomatic, unstable plaques compared with asymptomatic plaques and healthy carotid arteries in patients [40]. Increased FDG uptake was also observed in the atherosclerotic carotid arteries of rats [41], suggesting higher vascular glucose consumption in atherosclerosis. A study with carotid plaques from 159 atherosclerosis patients identified that the metabolite signature in high-risk plaques is consistent with increased glycolysis (with reduced levels of glucose but increased lactate) [42]. In parallel, glucose metabolism genes, including glycolysis and PPP genes, such as *SLC2A1*(glucose transporter type 1), *HK2*, *HK3*, *ALDOA*, *ENO1*, *TKT* (transketolase), and *PGD* (6-phosphogluconate dehydrogenase), were higher in plaques from patients with symptoms compared to those without [42]. In 2023, mass spectrometry imaging at the spatial level revealed that pyruvic acid and lactic acid were distinctly located in the fibrous cap and necrotic core of stable plaques, respectively [43]. Another study demonstrated significant heterogeneity in glucose metabolism among VSMCs, with glucose utilization varying based on their proliferative state and proximity to atherosclerotic plaques [44]. These observations suggest that cellular glucose metabolism may play a crucial role in the development of atherosclerosis, highlighting the importance of glucose metabolism in VSMCs during atherogenesis.

However, the evidence is not yet definitive because of multiple limitations, including: (i) species- and strain-specific findings in pigeons that may not generalize to mammalian atherosclerosis [35–39]; (ii) reliance on enzyme activity or mRNA abundance as surrogates for flux (no ^13^C tracing) and measurements made in bulk arterial tissue rather than purified VSMCs [37–39,42–44]; (iii) FDG-PET and plaque metabolite signatures that reflect composite signals from inflammatory cells, endothelium, and hypoxic regions, so they are not VSMC-specific [40–42]; (iv) largely cross-sectional designs without control for disease stage (stable vs. unstable) or systemic metabolic status; (v) spatial MS imaging that is semi-quantitative and does not by itself establish pathway directionality of glucose carbon [43]; and (vi) a paucity of VSMC-specific genetic or pharmacologic perturbations directly linking glycolytic reprogramming to lesion initiation/progression *in vivo*. These caveats argue for cautious interpretation and motivate VSMC-focused, flux-resolved, and stage-aware studies to define causality.

Given that glucose entry is the first step of glucose metabolism, the transporter expression and activity are a logical starting point. Glucose transport is regulated by a family of proteins that facilitate the movement of glucose across cell membranes. In VSMCs, the predominant glucose transporter is glucose transporter type 1(GLUT1/*SLC2A1*) [45,46], although other GLUT isoforms, such as GLUT4, may also be expressed in different kinds of smooth muscle cells under certain conditions (e.g., insulin-stimulated glucose uptake) [47,48]. It has been demonstrated that the GLUT1 protein level is strongly correlated with the VSMC phenotype in atherosclerosis and restenosis. GLUT1 protein and intracellular glucose levels were also increased in isolated neointimal tissues compared to those in the media after balloon injury [49]. VSMCs in proximity to atherosclerosis lesions express increased levels of the glucose transporter GLUT1 [50]. This indicates a link between GLUT1-mediated VSMC glucose metabolism and atherosclerosis. GLUT1 overexpression specifically in VSMCs enhanced the inflammatory phenotype of the artery wall through increased VSMC glycolysis and the polyol pathway. It stimulated monocyte recruitment and macrophage accumulation, ultimately accelerating atherosclerosis in a mouse model of metabolic syndrome [50], providing a direct *in vivo* evidence of the role of GLUT1 in atherosclerosis. However, the proposed TNF-α/GLUT1/glycolysis/polyol/CCL2/monocyte recruitment mechanism relied mainly on *in-vitro* inhibition with 2-deoxyglucose and aldose-reductase inhibitors plus transcriptional readouts; *in vivo* polyol dependence and glucose-carbon partitioning (e.g., ^13^C tracing) were not tested, and the study used an overexpression model without complementary VSMC-specific loss-of-function to establish necessity. Another study also found that overexpression of GLUT1 in VSMCs impaired vascular contractility and accelerated a proinflammatory, neutrophil-rich lesion, resulting in medial hypertrophy in response to wire injury. And this effect was considered to be associated with increased flux through PPP [20]. However, the causal role of PPP flux and VSMC GLUT1 per se remains unresolved: PPP “activation” was inferred indirectly from increased haptoglobin and GSH/total GSH rather than demonstrated by VSMC-specific flux assays. Thus, the data show association with GLUT1 overexpression rather than direct proof of PPP-dependent mechanisms. In summary, the role of GLUT1-mediated glucose entry in governing VSMC function during atherosclerosis and restenosis remains unclear and context-dependent. Resolving this will require VSMC-targeted knockout/knockdown, control for transporter redundancy, and *in vivo* flux assays rather than surrogate endpoints.

In addition to glucose transport, many glycolytic enzymes in VSMCs were also studied for their involvement in atherosclerosis and restenosis. Hexokinase, converting glucose into glucose-6-phosphate, is the first rate-limiting enzyme in glucose metabolism (Fig. 1). Docherty *et al*. reported elevated HK2 mRNA and protein in the fibrous cap of human plaques [51], consistent with a role for HK2 at the VSMC-rich interface; however, because caps contain mixed cell types (VSMC-, endothelial-, and macrophage-derived), these data implicate HK2 in atherosclerosis but do not establish VSMC specificity or causality. Increased HK1 expression and protein O-GlcNAcylation were observed in c-Kit^+^-derived cells during VSMC differentiation in an allograft model [52], suggesting a possible role; however, the evidence is largely correlative, lacking lineage-restricted HK1/OGT perturbation or flux-resolved assays could confound causality for neointima formation. Platelet-derived growth factor (PDGF), a well-known growth factor to promote VSMC phenotypic change, has been reported to increase HK2 protein expression and glycolysis evidenced by increased extracellular acidification rate (ECAR), and inhibition of hexokinase with inhibitor and siRNA reduced the migration of VSMCs *in vitro* [53]. However, *in vivo* validation is lacking in this study. Thus, the role of hexokinase in VSMC-driven atherogenesis and restenosis remains suggestive rather than proven.

In the regulation of glycolytic flux, PFK1-mediated catalysis of fructose-6-phosphate (F6P) to fructose-1,6-bisphosphate (F1,6P2) is one of the three rate-limiting checkpoints. Importantly, PFK-1 is activated by its allosteric activator, fructose-2,6-bisphosphate (F2,6P2), and the latter is synthesized by 6-phosphofructo-2-kinase/fructose-2,6-bisphosphatase (PFKFB). Mechanical stretch was reported to decrease fructose-6-phosphate (F6P) while increasing fructose-1,6-bisphosphate (F1,6P2), accompanied by reduced ubiquitination and enhanced catalytic activity of PFK1. These changes correlated with increased VSMC proliferation and migration *in vitro*, suggesting an association between PFK1-driven glycolysis and phenotypic switching [54]. Consistently, the same study showed that pharmacological inhibition of PFKFB3, an upstream activator of PFK1, suppressed VSMC proliferation and migration in vitro and alleviated neointimal hyperplasia in a mouse vein graft model [54]. However, direct evidence for a causal role of PFK1 in VSMCs is lacking. Different isoforms of PFKFB are localized in various tissues, with isoform 3 (PFKFB3) expressed in vascular cells and leukocytes, PFKFB1 in hepatocytes, PFKFB2 in cardiomyocytes, and PFKFB4 in testis [55–58]. Of these isoforms, PFKFB3 has the highest kinase activity, with a kinase/phosphatase activity ratio of 740:1 (Fig. 1) [55–58]. Several studies have demonstrated the role of PFKFB3-mediated glycolysis in vascular cells, including endothelial cells and macrophages, in the development of atherosclerosis [59–62]. In VSMCs, a recent study reported that inhibition of PFKFB3 with inhibitor PFK158 suppressed neointima formation induced by vascular injury. Mechanistically, they demonstrated that PFKFB3-mediated glycolysis provided acetyl-CoA as a fuel to facilitate fatty acid synthesis and VSMC phenotypic switching *in vitro* [63]. Additionally, a KLF4-mediated metabolic switch to glycolysis, driven by upregulation of PFKFB3, has been shown to be essential for KLF4-induced phenotypic changes of VSMCs in atherosclerotic lesions. Pharmacological inhibition of PFKFB3 with 2-DG or PFK15 suppressed the KLF4-driven transition of VSMCs into plasmacytoid dendritic cell (pDC)-like cells *in vitro* [64], underscoring the importance of PFKFB3-dependent glycolysis in this process. Nevertheless, no study has directly examined the role of VSMC-specific PFKFB3 in atherosclerosis *in vivo*, and thus its precise contribution remains unclear.

GAPDH, long treated as a “housekeeping” glycolytic enzyme, is dynamically regulated in VSMCs under atherogenic stress. In cultured VSMCs, oxidized low-density lipoprotein (oxLDL) lowers GAPDH expression/activity, cutting glucose utilization by ~65 % and ATP by ~80 %, consistent with a choke point at the triose-phosphate step and an energy-stress phenotype [65]. *In vivo*, smooth muscle restricted GAPDH overexpression reportedly reduces atherosclerosis in mice, although this evidence is from a meeting abstract, and the operative mechanism remains undefined [66]. In human atherosclerotic plaques, GAPDH has been observed to accumulate in the nucleus of dedifferentiated VSMCs, implying functions beyond its glycolytic activity [67]. Indeed, GAPDH is involved in several non-glycolytic processes, such as binding to nucleic acids and telomeres, regulating gene transcription, and modulating apoptosis [68]. These non-metabolic roles are collectively referred to as the non-glycolytic functions of the essential glycolytic enzyme. Supporting this, Hou et al. found that nuclear GAPDH interacted with DNA repair enzymes to protect VSMC against apoptosis [69]. Together, these observations suggest a compartment-specific model in which cytosolic GAPDH sustains glycolytic ATP/redox homeostasis, whereas nuclear GAPDH scaffolds DNA repair and transcriptional programs that influence VSMC survival and phenotype. The net impact on atherogenesis likely depends on context, such as oxLDL burden, oxidative stress, phenotypic state. Crucially, whether GAPDH’s atheroprotective signal derives primarily from glycolytic flux versus its nuclear non-glycolytic functions remains unresolved.

Another key glycolytic enzyme, Phosphoglycerate kinase 1 (PGK1), catalyzes the conversion of 1,3-diphosphoglycerate to 3-phosphoglycerate, producing the first ATP molecule in glycolysis (Fig. 1). PGK1 knockdown has been shown to decrease ADP/ATP ratio, suppress VSMC proliferation and migration significantly, and reduce neointima hyperplasia [70]. However, the study did not directly assess glycolytic flux, leaving it unclear whether the effects were due to impaired ATP generation, altered redox balance, or other downstream signaling. Moreover, reliance on knockdown rather than genetic models raises concerns about specificity.

Further downstream in the glycolytic pathway, pyruvate kinase can convert phosphoenolpyruvate (PEP) into pyruvate, which controls the rate-limiting step of aerobic glycolysis by either producing lactate or supplying acetyl-CoA for oxidative phosphorylation (OXPHOS) (Fig. 1). Two isoforms of the pyruvate kinase M gene exist: PKM1 (pyruvate kinase muscle isozyme 1) and PKM2 (pyruvate kinase muscle isozyme 2). These isoforms regulate the switch between glycolysis and OXPHOS. PKM1 is predominantly expressed in energy-demanding cells like brain and muscle cells [71], whereas PKM2 is found in highly proliferative cells such as tumor cells and synthetic VSMCs [71,72]. Although direct head-to-head metabolic tracing is limited, evidence suggests that PKM1, when present in cells lacking PKM2, tends to channel pyruvate into mitochondrial oxidation (OXPHOS) rather than lactate production. In contrast, PKM2’s dynamic regulatory state (dimer/tetramer equilibrium or association with glycolytic enzyme complexes) may favor routing of pyruvate toward lactate or biosynthetic branch pathways under proliferative conditions [73,74]. The atherogenic stimulus factor, oxLDL, upregulates and promotes glucose uptake, lactate and ATP production, and extracellular acidification rate (ECAR) in VSMCs *in vitro*. This process is dependent on the upregulation of pyruvate kinase isoform M2 (PKM2) [75]. Notably, oxLDL stimulation does not alter the oxygen consumption rate [75], suggesting a metabolic shift that favors aerobic glycolysis. Pharmacologic PKM2 inhibition (shikonin) blunts these oxLDL-induced changes, suppresses VSMC proliferation and migration, and delays atherosclerosis in *ApoE*^−/−^ mice, supporting a causal glycolytic role for PKM2 in this context [75]. Another genetic and pharmacological experiments indicate that PKM2 drives VSMC activation through dual mechanisms. On the glycolytic side, SMC-specific *PKM2* deletion reduces neointimal hyperplasia *in vivo* and, in PDGF-BB-stimulated SMCs, lowers lactate release and ECAR while attenuating ERK/mTOR/STAT3 signaling, consistent with diminished glycolytic drive and mitogenic signaling. On the non-glycolytic side, forcing PKM2 into its tetrameric state with ML265 prevents nuclear translocation and suppresses PDGF-BB-induced proliferation, migration, and phenotypic switching, while also disrupting PKM2’s interactions with STAT3/β-catenin, reducing STAT3 phosphorylation, decreasing PKM2 occupancy at the MEK5 promoter, and down-regulating MEK5, cyclin D1, GLUT1, and LDHA [72]. Taken together, the paper attributes the anti-neointimal effect to an integrated consequence of reduced glycolytic output and blockade of PKM2’s nuclear co-activator functions in VSMCs. Upstream splicing control further ties PKM2 to VSMC phenotype. PHB2 preserves the contractile state by binding hnRNPA1 to counteract PKM alternative splicing. PHB2 loss increases PKM2, boosts glycolysis, represses contractile markers, and aggravates neointima *in vivo* [76]. Similarly, LKB1 restrains PTBP1-dependent PKM splicing. LKB1 loss elevates the PKM2/PKM1 ratio, shifts metabolism toward aerobic glycolysis and VSMC plasticity, and is rescued by the PKM2 activator TEPP-46 [77]. Together, these studies position PKM2 as a dual-mode driver, via glycolysis and nuclear signaling, of VSMC phenotypic switching and vascular remodeling.

LDHA, which catalyzes the conversion of pyruvate to lactate (Fig. 1), is upregulated in neointimal VSMCs, indicating a central role in sustaining the glycolytic phenotype during vascular remodeling. Inhibition of LDHA reduces growth factor-stimulated glycolysis and suppresses VSMC proliferation and migration *in vitro* [78], establishing its importance for glycolytic energy supply. However, recent work has revealed that LDHA also influences VSMC behavior through both glycolytic and non-glycolytic mechanisms. Chen *et al*. show that LDHA crotonylation enhances lactate production and VSMC growth, and LDHA mono-ubiquitination induces the translocation of LDHA into mitochondria, which promotes VSMC proliferation and migration even beyond its catalytic role [79]. These findings broaden the view of LDHA from a terminal glycolytic enzyme to a multifaceted regulator.

The PPP is critical because it not only generates pentose phosphates to support nucleic acid synthesis, but also provides NADPH, which is required for both the synthesis of fatty acids and cell survival under stress conditions [80]. Glucose-6-phosphate dehydrogenase (G6PD) is the rate-limiting enzyme of the PPP (Fig. 1). Increased glucose flux through the PPP, enhanced G6PD activity, and elevated NADPH production have been observed in VSMCs under metabolic syndrome or during neointimal formation [81–83], indicating the potential role of G6PD-mediated PPP metabolic pathway in the pathological process of VSMCs. Indeed, pharmacological or genetic inhibition of G6PD activity increased expression of VSMC-restricted genes and suppressed transcriptomic and metabolic reprogramming in vascular tissues and concurrently reduced occlusive growth within coronary arteries in rats with metabolic syndrome [82]. In addition to its glycolytic effect, the non-metabolic role of G6PD in the regulation of VSMC function has been reported [84–86]. Currently, no study investigates the role of G6PD in VSMC specifically in atherosclerosis and restenosis using genetically modified mice *in vivo*.

HBP has been found to play an important role in atherosclerosis through O-GlcNAc modification in VSMCs. Early evidence demonstrated elevated O-GlcNAc levels in atherosclerotic plaques of diabetic patients [87]. And protective interventions that alleviated atherosclerosis were associated with reduced O-GlcNAcylation [88], suggesting a causal relationship. *In vitro*, high glucose activates HBP flux and increases O-GlcNAcylation, which in turn upregulates thrombospondin-1 (TSP-1), a potent proatherogenic protein that drives VSMC proliferation [89]. Similarly, enhanced O-GlcNAcylation has been directly linked to VSMC proliferation under hyperglycemic conditions, and its suppression attenuates this response [90]. Genetic models provide stronger evidence: VSMC-specific inducible deletion of O-GlcNAc transferase (OGT, regulator of O-GlcNAc signaling) prevented atherosclerosis in *Apoe*^−/−^ mice accompanied by decreased inflammatory and proliferative signals in VSMCs [91], underscoring a causal role for O-GlcNAc signaling in disease progression. Interestingly, constitutive SMC and cardiomyocyte deletion of *Ogt* or inhibition of OGT reduced arterial contractility [92,93], indicating that (i) O-GlcNAcylation regulates not only proliferative and inflammatory signaling but also contractile function; (ii) O-GlcNAcylation-regulated VSMC function may be context-dependent. In clinical settings, increased O-GlcNAcylation of specific protein targets has been linked to adverse vascular remodeling and vein graft failure in type 2 diabetes [94]. In summary, the prevailing concept is that increased O-GlcNAc modification in VSMCs contributes to the progression of atherosclerosis. However, current studies primarily demonstrate association or broad OGT dependence. It is unclear how elevated O-GlcNAc in VSMCs leads to a proliferative and atherogenic phenotype.

### 4.2. Glucose metabolism in pulmonary artery hypertension

Pulmonary artery smooth muscle cell (PASMC) hyperproliferation is a defining feature of pulmonary arterial hypertension (PAH), and accumulating evidence links this growth phenotype to a profound reprogramming of glucose metabolism. In monocrotaline (MCT) rat models, GLUT1 and HK1 are upregulated in PASMCs and endothelial cells, which corresponds with elevated FDG-PET uptake in the lung vasculature, a clinical hallmark of PAH [95]. PDGF, a key growth factor in PAH, promotes PASMC proliferation accompanied by increased lactate production and LDH, GLUT1, and GLUT4 expression, but decreased ATP production and pyruvate dehydrogenase (PDH) expression, suggesting a metabolic shift to aerobic glycolysis in PAH [96]. The molecular mechanism underlying the metabolic shift toward increased glycolysis in PASMCs during PAH is not fully understood.

Based on evidence of a glycolytic shift in PAH, multiple studies have examined the contribution of specific glycolytic enzymes to PASMC pathology. GLUT1 overexpression has been found to prevent VSMC apoptosis under hypoxic conditions [97,98]. HK2 was found to be increased in hypoxia-induced PASMCs and PASMCs from MCT-induced PAH rats [99,100]. Inhibition of HK2 can significantly suppress the proliferation of PASMCs and attenuate pulmonary hypertension through the inhibition of glycolysis [99,101]. These data suggest that upregulated glucose entry and phosphorylation are key to PASMC survival and growth under stress. Further downstream, PFKFB3, a rate-limiting regulator of glycolysis, is elevated in PAH. Its product, fructose-2, 6-bisphosphate, increases lactate output that in turn drives collagen synthesis and PASMC proliferation. VSMC-specific *Pfkfb3* deletion markedly attenuates PAH *in vivo* [102], providing strong causal evidence that glycolytic reprogramming fuels vascular remodeling.

Other glycolytic enzymes have also been implicated. ENO1 upregulation promotes the metabolic switch from oxidative phosphorylation to glycolysis, and its inhibition restores mitochondrial function and reverses PAH in mice [103,104]. PKM2 is likewise increased in lung tissue from experimental PAH models [105,106], suggesting a role for PKM2-mediated glycolysis in disease progression. Consistently, Li *et al*. reported that shikonin protected against MCT-induced PAH in rats by inhibiting PKM2, an effect associated with reduced glycolysis [106]. In contrast, Guo *et al*. found that reactive oxygen species (ROS)-mediated inhibition of PKM2 contributed to PAH progression, proposing that this inhibition redirected carbon into the PPP to support nucleotide synthesis and redox balance in PASMCs [107]. However, their evidence was largely correlative rather than causal. Adding further complexity, Zhang *et al*. demonstrated that PKM2 phosphorylation drives PASMC proliferation and migration via its protein kinase activity, independent of glycolysis [108]. Collectively, these studies implicate PKM2 in PASMCs of PAH but underscore the need for genetic approaches to clarify its precise metabolic and non-metabolic functions.

At the pyruvate node, pyruvate dehydrogenase kinase (PDK) also plays an important role in glycolysis by inhibiting PDH, which converts pyruvate to Acetyl-CoA, thereby redirecting pyruvate toward lactate and promoting aerobic glycolysis (Fig. 1) [109]. A study showed that PDK1 inhibition attenuated PASMC proliferation by regulating aerobic glycolysis *in vitro*[96]. Increased PDK4 expression was also involved in PASMC proliferation in hypoxia [110]. These studies indicate that PDK may be a promising target for PAH given its role in glycolytic regulation in PASMCs. Additionally, lactate itself has been shown to promote PASMC proliferation and migration, while LDHA inhibition or knockdown reduced lactate levels, suppressed hypoxia-induced proliferation and migration, and attenuated pulmonary hypertension [111], supporting the concept that excessive glycolytic flux and lactate accumulation directly contribute to PASMC-driven vascular remodeling in PAH.

Even though increased glycolysis is well established in PAH, evidence from patient samples suggests greater metabolic heterogeneity. Zhao *et al*. reported no detectable increase in glycolysis or lactate levels in the lungs of advanced PAH patients through metabolomics and microarray analysis [112]. But instead, high levels of glucose, sorbitol, and fructose are observed in the lungs of patients with PAH [112]. This raises the possibility that PPP activation, rather than glycolysis, may support vascular remodeling in certain disease contexts. Indeed, Boehme *et al*. found that PPP activity was elevated in hyperproliferative PASMCs at the early stage of PAH in a lamb model [113]. Mechanistically, the rate-limiting enzyme G6PD is upregulated under hypoxia and promotes PASMC proliferation *in vitro*, while its inhibition alleviates vascular remodeling in rat models of moderate and severe PAH [114]. In addition, G6PD inhibition restored the contractile phenotype [115] and reduced inflammatory signaling [116] in the pulmonary arteries. Finally, *de novo* purine synthesis, which depends on PPP-derived ribose-5-phosphate, has been shown to be essential for PASMC proliferation in PAH [18], further linking PPP flux to pathogenic vascular growth. Together, these studies suggest that the PPP provides both metabolic substrates and signaling inputs that complement or substitute for glycolysis, though its precise contribution to human PAH remains incompletely defined.

The contribution of the hexosamine biosynthetic pathway to PAH pathogenesis is only beginning to be understood. Protein O-GlcNAcylation, the major output of HBP flux, has been implicated in multiple aspects of the PAH, including endothelial angiogenesis, endothelial dysfunction, and right ventricular function in PAH [117–120]. In idiopathic PAH, Barnes *et al*. demonstrated that enhanced HBP flux augments global O-GlcNAcylation, which in turn promotes PASMC proliferation and correlates with faster clinical progression [121]. Thus, while early evidence positions HBP-mediated O-GlcNAcylation as a potential pathogenic pathway in PAH, more work is required to establish causality and to determine whether targeting OGT or O-GlcNAc cycling can selectively suppress vascular remodeling without impairing essential cellular functions.

### 4.3. Glucose metabolism in calcification

Human and clinical signals point to a metabolic contribution to calcification, but they are associative and likely reflect underlying flux changes rather than glucose entry alone. For example, a GLUT1 *Xbal* gene polymorphism, which is linked to the changes in intracellular glucose concentration, correlates with vascular calcification in nondiabetic uremic patients [122]. And plasma lactate positively associates with calcification burden in clinical cohorts [123,124]. These observations are compatible with a state of heightened glucose metabolism drive in the calcifying vasculature, but they do not establish causality or the operative cell type.

At the cellular level, multiple studies converge on increased glycolysis as a driver of osteogenic trans-differentiation in VSMCs. Osteogenic matrix cues such as bone Gla protein/osteocalcin upregulate HIF-1α-dependent glycolysis and perturb Wnt signaling, which is sufficient to induce cartilage/vascular calcification and arterial osteogenic programs [125,126]. Additionally, PFKFB3 expression, LDH level, and lactate content significantly increased during calcification of VSMCs [127,128]. Functionally, PFKFB3 inhibition (pharmacologic or knockdown) attenuates calcification *in vitro* and *in vivo*, and mechanistically PFKFB3-driven glycolysis promotes the osteogenic switch at least partly via altered FoxO3 expression and lactate production [128]. These data support a model in which rate-controlling glycolysis, such as PFKFB3, provides both bioenergetic support and signaling metabolites that bias chromatin/transcription toward an osteogenic state. The limitation is that most experiments used cultured cells and inhibitors with potential off-targets. VSMC-specific genetic perturbation and flux tracing would strengthen the causal link.

A complementary regulator is PDK4, which regulates cellular glycolysis by inhibiting PDH and shunting pyruvate away from mitochondria. PDK4 has also been indicated to be closely related to vascular calcification. PDK4 was found to be upregulated in calcifying VSMCs and calcified vessels of patients with atherosclerosis [129,130]. Inhibition of PDK4 (genetic or pharmacologic) reduces calcification in phosphate-treated VSMCs/aortic rings and in vitamin D3-treated mice [129]. Mechanistically, evidence diverges: one study reports a SMAD1/5/8 phosphorylation mechanism that appears glycolysis-independent [129], whereas others implicate suppressed autophagy and metabolic reprogramming toward glycolysis, with AGE-HIF-1α/PDK4 activation accelerating calcification while suppressing glucose oxidation [130,131]. Taken together, PDK4 likely promotes calcification through both glycolytic and signaling/autophagy pathways, with context (stimulus, duration, model) determining which axis dominates.

Extracellular matrix cues can also reprogram VSMC metabolism upstream. Periostin enhances glycolysis via PPARγ-related transcriptional changes, promotes the osteoblastic phenotype, and glycolysis inhibition blunts periostin-induced calcification [123]. This suggests ECM remodeling not just as a consequence of calcification but as a metabolic trigger that tunes glycolytic flux and fate decisions in VSMCs.

Beyond glycolysis, HBP and O-GlcNAcylation constitute a nutrient-sensing layer that directly activates osteogenic signaling. Studies indicated that increased vascular calcification in human and mouse diabetic vascular lesions is associated with increased O-GlcNAcylation via the hexosamine biosynthesis pathway [87,132–135]. Notably, Studies demonstrated a causative effect of GlcNAcylation-mediated activation of protein kinase B (AKT) to drive calcification in diabetes models [134]. And O-GlcNAcylation of runt-related transcription factor 2 (RUNX2) enhances osteogenic transcription in VSMCs [135]. Perturbations in intracellular Ca^2^^+^ homeostasis further amplify O-GlcNAc and vascular stiffness in diabetes [135], suggesting cross-talk between ionic signaling and glucose-sensing O-GlcNAc modification. These studies imply that VSMC-specific *in vivo* tests of HBP and OGT/OGA remain a clear gap.

### 4.4. Glucose metabolism in aneurysm and dissection

Aortic aneurysm is the second prevalent aortic disease following atherosclerosis [136], which is characterized by VSMC dysfunction [137]. The studies of the relationship between glucose metabolism and aneurysms are limited. Diabetes mellitus (DM) has been proven to contribute to multiple comorbidities that are risk factors for aneurysms. However, epidemiological studies reveal a negative correlation between hyperglycemia and aneurysm incidence [138–140]. Several mechanisms may underlie this intriguing paradox: Chronic hyperglycemia promotes the accumulation of advanced glycation end-products (AGEs), which increase collagen and elastin cross-linking in the aortic wall and may enhance wall stiffness and resistance to dilation [141,142]. Diabetes has also been linked to reduced activity of matrix metalloproteinases such as MMP-2 and MMP-9, thereby limiting elastin and collagen degradation that drives aneurysm expansion [142,143]. In addition, the pleiotropic vascular effects of antidiabetic medications such as metformin or DPP-4 inhibitors, may contribute to the restraint of aneurysm growth [144]. Although the precise mechanisms remain incompletely resolved, these findings provide plausible explanations for the epidemiological paradox and highlight how glucose metabolism and its downstream consequences can differentially influence vascular pathologies. It raises the question of the cellular role of glucose metabolism in aneurysms, especially in VSMCs.

There is a study that showed serum lactate levels were markedly elevated in the aortic aneurysm/dissection patients [145]. Both clinical and experimental aneurysms showed increased glucose uptake through GLUT1 expression [146,147]. And pharmacological inhibition of glycolysis in myeloid cells can alleviate aneurysms in the mouse model [147], suggesting the critical role of glycolysis in the development of aneurysms. However, these studies did not investigate the role of VSMC glycolysis in this process. A recent study reported activation of glycolysis and pyruvate metabolism pathways in VSMCs from human aortic dissection samples based on single-cell sequencing analysis, indicating that the evidence is currently limited to the transcriptional level [148]. Scientists have found that Ang II stimulation increases GLUT1 expression and enhances glucose uptake in cultured VSMCs [149,150]. However, VSMC GLUT1 deficiency did not alter Ang II-induced AAA development or rupture in a mouse model [151], suggesting that glucose uptake in VSMCs may not be rate-limiting for aneurysm development, or that alternative transporters can compensate. In contrast, downstream glycolytic regulation appears more critical. ENO2, a glycolytic enzyme, was markedly upregulated in human aortic dissection tissue, and VSMC-specific knockdown of ENO2 attenuated dissection formation by restoring autophagy and cellular homeostasis [152], highlighting a direct mechanistic link between glycolysis and VSMC stress responses. Similarly, mitochondrial dysfunction has been shown to enhance glycolysis in VSMCs, promoting aneurysm pathogenesis through impaired oxidative metabolism and increased metabolic reliance on glycolysis [153]. Moreover, the transcription factor TCF7L2 was recently identified as a regulator of VSMC glycolysis, where its deficiency mitigated AAA formation [154], suggesting that transcriptional control of metabolic flux contributes to aneurysm susceptibility. All the above studies suggest that the glucose metabolism of VSMC is intricate in aneurysm and dissection; further studies are required to explore this further.

Notably, PKM2 has recently been implicated in aneurysm and dissection through distinct mechanisms. Li *et al*. demonstrated that a newly discovered gene, March2 (membrane-associated RING finger protein 2), promotes PKM2 polymerization; March2 deficiency reduced PKM2 tetramerization, increased lactylation, and aggravated aortic aneurysm/dissection (AAD). Notably, pharmacological activation of PKM2 with TEPP-46 restored tetramerization, reduced lactate accumulation and was protective against AAD even in wild-type mice [145]. In contrast, Wang *et al*. identified glucose metabolism regulatory protein (GMRSP), a microprotein encoded by lncRNA H19, as a suppressor of PKM2 splicing and glycolysis. GMRSP overexpression preserved the contractile phenotype of VSMCs and protected against AD, whereas TEPP-46 increased ECAR and lactate production and abrogated the protective effect of overexpression of GMRSP, thereby exacerbating AAD under the GMRSP overexpression background. Interestingly, part of their data also showed that TEPP-46 did not significantly affect AAD in control mice [148]. These findings highlight a striking controversy. Several factors may account for the divergent results. First, although both studies used the β-aminopropionitrile (BAPN)-induced AAD model, the experimental designs differed: Li *et al*. administered TEPP-46 after disease induction, whereas Wang *et al*. treated mice concurrently with BAPN, which may differentially influence disease progression. Second, some discrepancies arise from the genetic background: Li *et al*. tested wild-type, whereas Wang *et al*. focused on GMRSP overexpression, a condition that already suppresses glycolysis, potentially altering how PKM2 activation manifests. Even so, the control groups across studies still revealed opposing outcomes, underscoring a deeper mechanistic inconsistency. Third, the most provocative conflict lies in the metabolic readouts. Wang’s study reported that TEPP-46 increased glycolysis (higher ECAR and lactate) in VSMCs, which runs counter to most current evidence showing that PKM2 activation enforces tetramerization, reduces glycolytic flux, and enhances oxidative phosphorylation (OXPHOS) in diverse systems [155–157]. Together, these results suggest that the metabolic consequences of PKM2 activation may be highly context-dependent, shaped by timing of intervention, genetic background, and upstream metabolic wiring. Future work using genetic mouse models, flux tracing, and side-by-side comparisons across experimental contexts will be critical to reconcile whether PKM2 activation and its effects on glycolysis are fundamentally protective or pathogenic in aneurysm disease.

### 4.5. Lactate and lactylation in vascular diseases

Lactate is the product of anaerobic glucose metabolism in VSMCs. It has long been considered a metabolic waste product. Although VSMCs exhibit high rates of glycolysis and produce substantial amounts of lactate under normoxic conditions, whether they can effectively utilize lactate as a metabolic fuel remains unresolved. Rising direct evidence from isotope-tracing studies indicates that lactate is taken up by cells through monocarboxylate transporters (MCTs) and oxidized in mitochondria, including neurons [158] and tumor cells [159,160]. The machinery required for lactate transport, including MCT1, MCT3, and MCT4, has been detected in VSMCs [161,162], suggesting a potential capacity for lactate uptake under certain conditions in VSMCs. However, definitive isotopic tracer studies demonstrating robust lactate oxidation in VSMCs are lacking, and current evidence points toward a predominant role of lactate as a signaling metabolite-promoting phenotypic switching, vascular calcification, and histone lactylation-rather than as a primary energy source in VSMCs.

Yang et al. demonstrated that lactate can promote the VSMC synthetic phenotype. And this process is lactate transporter-dependent [163]. It was also reported that lactate accelerated osteoblastic phenotype transition of VSMCs, which was associated with mitophagy and apoptosis [164–166]. These studies suggest that excess lactate is detrimental to VSMCs. However, some of these experiments use high (exogenous) lactate concentrations in culture, which may not reflect physiological levels. On the contrary, there is also a study indicating that inactivation of the lactate transporter, monocarboxylate transporter (MCT3), also promotes VSMC synthetic phenotype transition [162]. But they failed to demonstrate that the effect resulted from the dysfunction of lactate transport. Thus, these data suggest that the role of lactate or lactate transport in VSMC biology may be context-dependent and may vary by transporter subtype and disease state.

Moreover, lactylation, a post-translational modification, has been identified to modulate protein and gene expression in cells [167]. Mechanistically, glycolytic pyruvate is reduced to lactate by LDHA, expanding the intracellular lactate pool. A portion of this lactate is converted to lactyl-CoA, which serves as the acyl donor for histone lysine lactylation [167]. Acyltransferases such as p300 and HBO1 function as “writers” [167,168], while HDAC1–3 serve as “erasers” creating a dynamic system that links glycolytic flux directly to chromatin remodeling [169]. In parallel, the glyoxalase pathway can produce lactoyl-glutathione, giving rise to D-lactylation, further broadening the cellular lactylome [170].

Consistent with this, recent studies in VSMCs demonstrate that lactate-driven histone lactylation underlies key pathogenic transitions, though the strength of evidence varies. For example, tumor necrosis factor α (TNF-α) induced SOX10 (Sex-determining region Y (SRY)-related HMG-box gene 10) lactylation, which was reported to associate with macrophage-like VSMC transition in neointimal hyperplasia [171], but this was largely correlative and lacked direct demonstration that lactylation is required for the phenotypic change. In contrast, stronger causal evidence was provided by a study showing that TRAP1 (Tumor necrosis factor receptor-associated protein 1) upregulated glycolysis, leading to increased lactate accumulation, enhanced H4K12la via the histone lysine delactylase, which subsequently promoted VSMC senescence and atherosclerosis [172]. Similarly, one recent study showed that NR4A3 (nuclear receptor subfamily 4 group A member 3)-mediated histone lactylation promoted arterial calcification by enhancing glycolysis activity and increasing lactate production in VSMCs, which reveals a novel metabolome-epigenome signaling cascade mechanism with direct functional outcomes [173]. Additional studies have linked lactylation to vascular calcification, with elevated global lactylation in calcified VSMCs and H3K18la specifically implicated in diabetic calcification [174]. However, most of these remain associative. In aneurysm/dissection, lactylation (particularly H3K18la) was found to be increased in diseased aortic tissue and TNF-α-treated VSMCs, but again the causal contribution to pathogenesis was not demonstrated [145]. In PASMCs, lactate accumulation resulted in elevation of H3K18 and H4K5 lactylation and promoted PASMC proliferation [175]. Study also showed that the long noncoding RNA (LncRNA) UNC5B-AS1 can inhibit the inflammatory VSMCs transition through suppressing glycolysis mediated H3K18la in PAH [176], providing functional evidence for this axis. Here, we summarize the research on lactate and lactylation in VSMCs. The elaborate summary of the study of lactate and lactylation in cardiovascular disease is described in the relevant review paper [177].

Taken together, these studies highlight lactylation as a potentially important epigenetic link between glycolysis and VSMC dysfunction. However, the field is still emerging: while some experiments demonstrate causality through genetic or pharmacological manipulation of lactylation, others remain descriptive. Clarifying which lactylation events are true drivers versus secondary markers of disease will be critical for evaluating the therapeutic potential of targeting the lactatelactylation axis in VSMCs of vascular pathologies.

## 5. Molecular mechanisms linking glucose metabolism to vascular diseases

Hypoxia-inducible factor 1 (HIF-1) is a transcription factor that functions as a master regulator of oxygen homeostasis [178]. HIF-1 regulates angiogenesis and vascular remodeling by regulating glucose metabolism and redox homeostasis. HIF-1 increases the expression of several pro-glycolytic enzymes, such as HK2 [179] and PDK [180]. As a well-known inducer of glycolysis, HIF-1 is active in vascular diseases [181,182]. HIF-1α has been reported to regulate PDK4-mediated glucose metabolism in VSMC calcification [131]. Additionally, in pathological VSMCs, HIF-1α is activated to induce increased glycolysis, promoting proliferation through the targeting of PFKFB3 [63,96]. Another study also indicated that HIF-1α triggers the PASMC glycolysis switch and histone lactylation in PAH, which occurs through the targeting of PDK1 and PDK2 [175]. Lambert *et al*. demonstrated that inhibition of HIF-1, using a dominant-negative HIF-1α adenoviral construct, attenuated carotid artery post-injury remodeling in rats by promoting apoptosis and reducing the proliferation of VSMCs through targeting HK-2-mediated glycolysis [183].

The mammalian target of rapamycin (mTOR) is a central signaling molecule that responds to changes in nutrient levels and growth signals. mTOR functions in two distinct complexes, mTORC1 and mTORC2, which regulate cellular glucose metabolism by modulating the expression of related metabolic enzymes [184]. mTORC1 is a nutrient-sensitive complex that regulates glucose metabolism by controlling glycolytic enzymes and growth pathways. In VSMCs, mTORC1 has been shown to drive glycolysis and phenotypic switching during vascular remodeling: Pharmacological or genetic inhibition of mTORC1 suppressed the proliferation and migration of VSMCs in intimal hyperplasia [185–187]; *MTMR7* deficiency or *DDX17* deficiency suppressed VSMC phenotypic transition through reduced p62/mTORC1 or RHEB/mTORC1 signaling, respectively. By contrast, mTORC2 is less responsive to nutrient status but acts as an upstream positive regulator of glycolysis and cell growth under stress conditions: Goncharov *et al*. showed that mTORC2, but not mTORC1, promotes PASMC glycolysis and proliferation by targeting AMPK, contributing to pulmonary arterial hypertension (PAH) [188]. Other studies have linked mTOR signaling more broadly to glycolytic control in PASMCs: Platelet-derived TGF-β1 was shown to enhance aerobic glycolysis via PKM2 upregulation in an mTOR-dependent manner [189]; Global metabolomic analyses revealed that mTOR signaling extensively remodels PASMC glucose metabolism in PAH [190]. Notably, these studies did not distinguish between mTORC1 and mTORC2, underscoring the need for further work to parse complex-specific roles in PASMCs. In summary, the available evidence suggests that both complexes contribute to vascular disease via the regulation of glucose metabolism, making mTOR signaling an attractive therapeutic target [191,192].

The transforming growth factor-β (TGF-β) signaling plays a critical role in the development and tissue homeostasis, and it can actively alter glucose metabolism in diverse cell types [193]. There is evidence indicating that TGF-β signaling can regulate VSMC glucose metabolism and cell proliferation via targeting peroxisome proliferator-activated receptor gamma (PPARγ) [194]. Krüppel-like factor 4 (KLF4), as a molecular fate switch [6,195], plays a key role in regulating glycolytic shift during VSMC phenotypic switching in atherosclerosis [64]. The study also suggested the therapeutic potential of targeting KLF4-dependent phenotypic modulation of VSMC in atherosclerosis [6].

## 6. Translational opportunities and challenges

There is a great need for efficient and specific therapeutics to treat life-threatening vascular diseases. As discussed above, pathological VSMCs reprogram their glucose metabolism during vascular diseases, which raises the question of whether targeting VSMC glucose metabolism should be considered in treatment strategies. Currently, as the technical and conceptual advancements required to unravel the intricate details of VSMC metabolism entirely are only beginning to surface, targeting VSMC glucose metabolism therapeutically remains in its early stage. Nevertheless, some studies have still presented compelling proof of this concept (Table 2). 3-(3-pyridinyl)-1-(4-pyridinyl)-2-propen-1-one (3PO), a specific PFKFB3 inhibitor, has been demonstrated to attenuate the progression of MCT-induced or Sugen/hypoxia-induced PAH in rats, partially through regulating VSMCs’ glycolysis [102,196]. Additionally, another PFKFB3 inhibitor, PFK15 (1-(4-pyridinyl)-3-(2-quinolinyl)-2-propen-1-one), is reported to ameliorate vascular calcification in Vitamin D3-overloaded mice *in vivo* and attenuate high phosphate (Pi)-induced VSMC calcification *in vitro* [128]. Moreover, Shikonin (SKN), a specific inhibitor of PKM2, was found to inhibit oxLDL-induced proliferation and migration in VSMCs and to delay atherosclerosis progression in *ApoE*^*−*/−^ mice [75]. An inhibitor of HK2, the small molecule 3-bromopyruvate (3-BrPA), also effectively reverses vascular remodeling in hypoxia-induced PAH rats via suppressing glycolysis [99,101]. These studies indicate the promising translational potential to target glycolysis in VSMCs. In addition to glycolysis, targeting G6PD-mediated PPP is another strategy. It has been demonstrated that G6PD inhibitor epiandrosterone (EPI) or dehydroepiandrosterone (DHEA) treatment reduces neointimal formation in the coronary artery and large artery elastance in metabolic syndrome rats and accentuates pulmonary hypertension via regulation of VSMC phenotype by metabolic reprogramming [82,114]. Furthermore, targeting the upstream regulator of glucose metabolism has also demonstrated effectiveness in research: administering the mTOR kinase inhibitor PP242 successfully reversed hypoxia-induced pulmonary vascular remodeling in rats [188]. Inhibition of HIF-1α with adenovirus prevented carotid artery post-injury remodeling in rats [183].

Despite these encouraging findings, most of the compounds used to date have limited specificity, potential off-target effects, and uncertain pharmacokinetic or toxicity profiles. Delivery challenges, especially for gene-based interventions such as HIF-1α constructs, also remain significant barriers (Table 2). Thus, while proof-of-concept studies highlight the feasibility of targeting VSMC metabolism, their translational readiness remains low to moderate.

The intricate and multifaceted role of VSMCs in vascular disease presents a landscape of challenges for targeting VSMC glucose metabolism to treat vascular diseases. First, deciphering the heterogeneous metabolism of VSMCs and selectively targeting the characterized VSMC subpopulation is a slow and long way to go. Recent single-cell and spatial studies already highlight that VSMCs comprise diverse substates-including contractile, synthetic, inflammatory, and osteogenic-like phenotypes-with distinct metabolic features. For example, imaging mass spectrometry has revealed focal enrichment of glucose utilization in VSMCs beneath atherosclerotic plaques [44], while enzyme histochemistry and spatial metabolomics of human lesions demonstrated region-specific heterogeneity of glycolytic and pentose phosphate pathway activity [43,197]. Single-cell RNA-seq has identified multiple SMC-derived clusters in both mouse and human lesions [198–200], including subclusters enriched for glycolytic gene signatures such as *ALDOA* [200], and recent integrative analyses further consolidated disease-relevant VSMC states [201]. Integrating single-cell RNA-seq with spatial metabolomics and isotope tracing would provide a concrete pipeline to resolve such metabolic subpopulations and define their functional dependencies. Additionally, the intervention that can precisely modulate VSMC metabolism while avoiding systemic adverse effects remains a significant hurdle. Moreover, optimizing the delivery of therapeutic agents, such as VSMC-targeted nanoparticles to VSMCs efficiently and safely is another challenge. Although difficult to achieve, restoring the abnormal glucose metabolism in VSMCs will still be crucial for the success of new therapeutic approaches.

## 7. Conclusion, discussion, and future directions

**Fig. 2.**
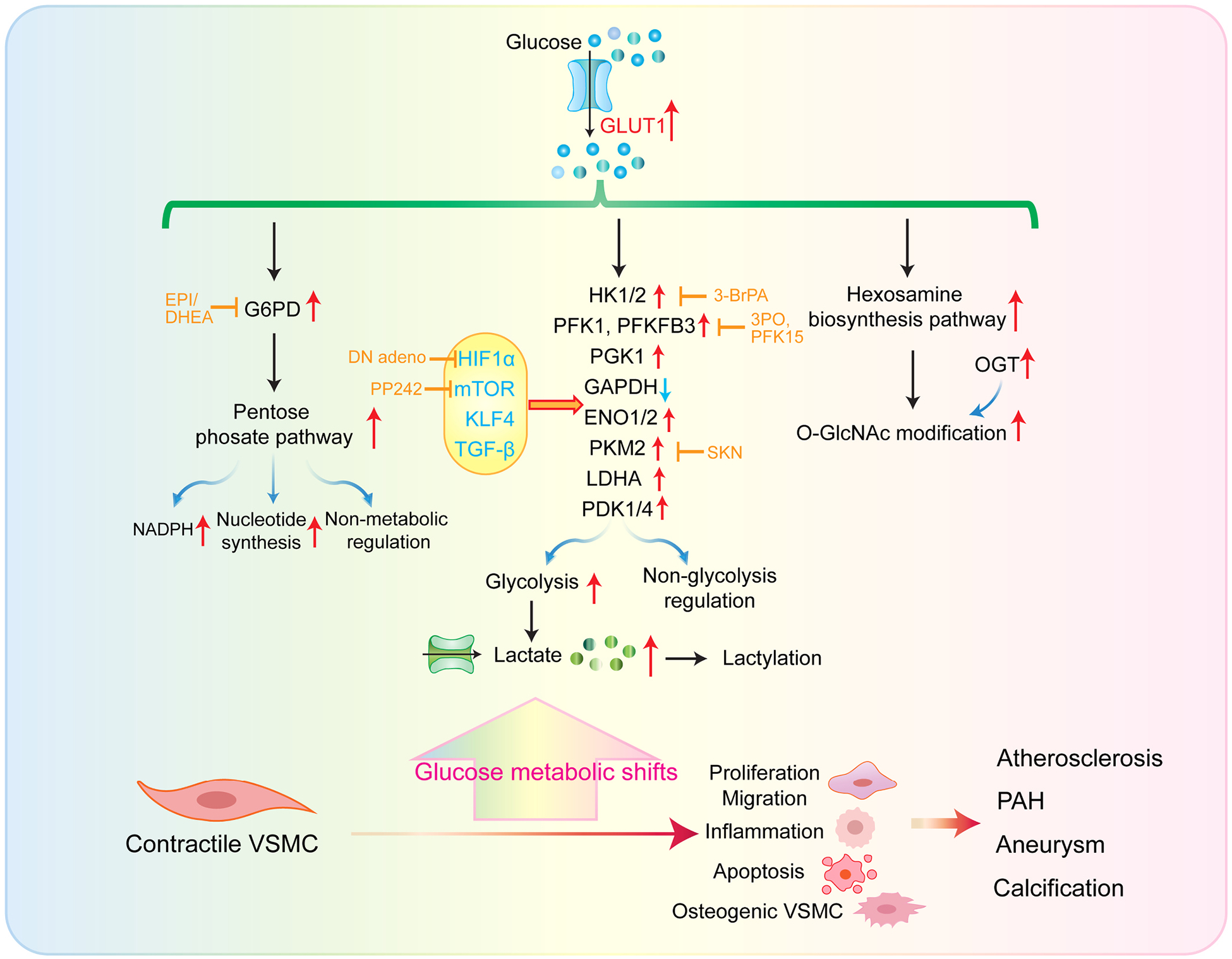
The glucose metabolic shifts in VSMCs in the context of vascular diseases. Scheme depicting glucose metabolic shifts in VSMCs and their pathological consequences. Increased glucose uptake via GLUT promotes enhanced glycolytic flux, the hexosamine biosynthesis pathway (HBP), and alterations in the pentose phosphate pathway (PPP). Key metabolic enzymes, including HK1/2, PFK1, PFKFB3, PGK1, ENO1/2, and LDHA, show upregulation, driving glycolysis and lactate production. These shifts are regulated by pathways involving HIF-1α, mTOR, KLF4, and TGF-β signaling, which influence metabolic reprogramming and cellular functions. Concurrently, HBP activity increases O-GlcNAc protein modification via OGT. PPP pathway is also upregulated via G6PD. The metabolic alterations lead to phenotypic changes in VSMCs, including increased proliferation, migration, inflammation, and osteogenic differentiation. These changes contribute to the progression of vascular diseases such as atherosclerosis, pulmonary arterial hypertension (PAH), aneurysm formation, and vascular calcification. Pharmacological inhibitors (e.g., 3PO, PFK15, SKN) targeting specific metabolic enzymes are indicated as potential therapeutic strategies to mitigate these pathological outcomes. HIF-1α, Hypoxia-inducible factor 1α; mTOR, mammalian target of rapamycin; KLF4, krüppel-like factor 4; TGF-β, transforming growth factor-β; 3PO, 3-(3-pyridinyl)-1-(4-pyridinyl)-2-propen-1-one; PFK15, 1-(4-pyridinyl)-3-(2-quinolinyl)-2-propen-1-one; SKN, Shikonin; 3-BrPA, 3-bromopyruvate; EPI, epiandrosterone; DHEA, dehydroepiandrosterone; DN adeno, dominant negative adenoviral construct.

This review underscores the central role of glucose metabolism in shaping vascular smooth muscle cell behavior and its impact on vascular disease progression, stimulating greater interest in this area of research. In summary, evidence to date indicates that diverse pathological stimuli-including growth factors, oxidative stress, hypoxia, and pro-calcific cues-induce metabolic shifts in VSMCs. These shifts reprogram glycolysis, the hexosamine biosynthetic pathway, and the pentose phosphate pathway, driving transitions from a contractile to proliferative, migratory, inflammatory, or osteogenic phenotypes. Enzymes such as GLUT1, HK2, PFKFB3, PKM2, G6PD, and OGT are emerging as candidate therapeutic targets (Fig. 2). However, the overall landscape of VSMC glucose metabolism remains only partially defined. It is highly warranted to characterize further the metabolic regulation linked to healthy VSMCs and diseased VSMCs.

Whether glucose metabolic reprogramming is a primary driver or a secondary consequence of VSMC phenotypic switching remains an unresolved question. Some experimental studies suggest a causal role, but the evidence is still limited, as noted earlier in this review. Conversely, classical phenotypic stimuli such as PDGF-BB, oxidized lipids, and osteogenic conditions reliably induce contractile-to-synthetic or osteogenic transitions that are accompanied by glucose metabolic shifts. Together, these observations imply that metabolic rewiring can function both as a driver and as a consequence of VSMC plasticity, with reciprocal feedback loops reinforcing disease-associated phenotypes. Future studies employing lineage-restricted metabolic perturbations and temporally resolved single-cell multi-omics will be crucial to disentangle causality and define the hierarchical order between metabolic changes and phenotypic fate decisions.

To advance this field, we propose a prioritized research agenda. In the near term (1–3 years), efforts should focus on systematically linking specific glucose-metabolic enzymes to VSMC phenotypic outcomes using standardized assays: metabolic flux analyses (^13^C-glucose or ^13^C-lactate tracing, ECAR/OCR), epigenetic readouts (histone lactylation, O-GlcNAcylation), and functional endpoints (proliferation, migration, calcification, apoptosis, and inflammatory cytokine release). In parallel, lineage-restricted *in vivo* models-such as smooth muscle-specific knockout or overexpression of glycolytic and auxiliary pathway enzymes-should be employed in atherosclerosis, restenosis, pulmonary hypertension, calcification, and aneurysm contexts to establish causal relationships. In the medium term (3–7 years), multi-omics technologies will be essential to capture the full regulatory complexity of VSMC metabolism. Transcriptomics can reveal cell-state-specific enzyme expression patterns, proteomics can define post-translational modifications and signaling crosstalk, and metabolomics can quantify pathway activity and flux. Integration of these datasets, especially with spatial transcriptomic and metabolomic profiling, will allow mapping of metabolic microenvironments within lesions and identification of disease-relevant VSMC sub-states.

Artificial intelligence (AI) will serve as a critical accelerator in this next phase. Machine-learning algorithms can harmonize multi-omics data across laboratories, minimize batch effects, and enable reproducible meta-analyses. Deep-learning approaches can mine high-dimensional datasets to uncover latent metabolic signatures predictive of VSMC phenotype transitions or vascular lesion stability. Predictive modeling can integrate omics, imaging modalities (e.g., PET, intravascular ultrasound, spatial metabolomics), and clinical data to stratify patients into metabolic “endotypes” with distinct therapeutic vulnerabilities. Moreover, AI-driven image analysis can standardize quantification of VSMC proliferation, lesion size, or calcification in both animal models and human tissues, thereby reducing inter-observer variability and facilitating cross-study comparisons. Finally, network-based AI tools can predict novel metabolic regulators and prioritize candidate drug targets, guiding translational studies and therapeutic development.

Together, this integrated roadmap-combining standardized experimental protocols, multi-omics discovery platforms, and AI-enabled integration and prediction-offers a concrete, testable path forward. It moves the field beyond descriptive associations toward a mechanistic, reproducible, and clinically translatable framework for understanding and targeting VSMC glucose metabolism in vascular disease.

## Figures and Tables

**Table 1:** Summary of major studies on glucose metabolism in VSMCs in vascular diseases.

Disease/model	Control nodes	Pathway	Species	Preparation	Primary finding	Perturbation and outcome	Evidence grade	Year/References
atherosclerosis	PFK, Aldolase	glycolysis	pigeon	aortas with adventitia removed	PFK, Aldolse enzyme activity↑ in WC compared SR		correlative *ex vivo*	1975 [37, 38]
restenosis	GLUT1	glucose	mouse	Isolated neointimal tissue	GLUT1 protein expression↑ (WB)	overexpression of GLUT1→apoptosis↓ in rat aortic VSMCs	mechanistic *in vitro*	2001 [49]
atherosclerosis	GLUT1	glycolysis, polyol	mouse	atherosclerotic lesions in the brachiocephalic artery	GLUT1 mRNA↑ in SMCs underlying lesions (*in situ* hybridization)	SMC GLUT1 overexpression → glycolysis and polyol intermediates↑ → monocyte recruitment↑→ atherosclerosis↑	genetic *in vivo*; correlative	2018 [50]
restenosis	GLUT1	PPP	mouse	SMC GLUT1 overexpression → PPP↑→vascular contractility↓, inflammation↑ → neointima↑			genetic *in vivo*; correlative	2011 [20]
AAA	GLUT1	glucose	mouse	SMC GLUT1 knockout → did not alter AAA			genetic *in vivo*	2025 [151]
atherosclerosis	HK2, GAPDH, LDH, PDK	glycolysis	human	dissected carotid arteries → *HK2* mRNA↑ in cap of plaque; carotid atherectomy specimens → staining of GAPDH, LDH, PDK, HK↑ in cap; cultured plaque VSMCs → ECAR↑			correlative	2018 [51]
PAH	HK2	glycolysis	rat	rat PASMCs in hypoxia	HK2 protein↑; lactate↑	HK2 inhibition (3-BrPA)→HK2↓, lactate↓→PASMC proliferation, migration↓, apoptosis↑→PAH↓	Pharmacological *in vivo*	2018 [99], 2019 [101]
restenosis	HK1	glycolysis, HBP	human, mouse	TGFβ treated c-Kit^+^ SMCs	HKprotein/activity↑; ECAR↑; protein O-GlcNAc↑	2-DG/HK1 siRNA/O-GlcNAc inhibitor → c-Kit^+^ SMCs↓→neointima↓	mechanistic *in vitro*	2019 [52]
restenosis	PFK1	glycolysis	human, mouse	VSMCs subjected mechanical stretch	F–1,6-P2↑, F6P↓; ECAR↑; PFK1 ubiquitination↓; PFK1 activity↑	PFKFB3 inhibitor (PFK15) →VSMC proliferation, migration↓→ neointima↓	correlative; pharmacological *in vivo*	2022 [54]
restenosis	PFKFB3	glycolysis	rat, mouse	PDGF treated rat VSMCs; mouse carotid arteries with wire injury;	PFKFB3 protein expression↑; ECAR↑	*In vitro*: si*PFKFB3*→VSMC proliferation, migration↓ *In vivo*: PFKFB3 inhibitor (PFK158)→ neointima↓	Pharmacological *in vivo*	2023 [63]
calcification	PFKFB3	glycolysis	human, mouse	calcified VSMCs and arteries	PFKFB3↑	*In vitro*: si*PFKFB3*→VSMC calcification↓ OE-*PFKFB3*→VSMC calcification↑ pyruvate/lactate→effect of siPFKFB3↓ *In vivo*: PFKFB3 inhibitor (PFK15)→vascular calcification↓	Pharmacological *in vivo*	2023 [128]
PAH	PFKFB3	glycolysis	human, mouse, rat	lung and PASMCs from IPAH patients; rat and mouse PAH lung	PFKFB3 protein↑; lactate↑	*In vitro*: si*PFKFB3*/3PO→collagen synthesis↓→VSMC proliferation↓, apoptosis↑ *In vivo*: SMC-*Pfkfb3*^KO^, PFKFB3 inhibitor (3PO)→PAH↓	2019 [102]	
calcification	PDK4	non-glycolysis	human, mouse	calcified VSMCs and arteries	PDK4 expression, activity↑	*In vitro*: PDK interacts with SMAD *Ex vivo*: PDK inhibitor→calcification↓ *In vivo*: *PDK4*^−/−^→vascular calcification↓	genetic *in vivo*; pharmacological *ex vivo*	2015 [129]
atherosclerosis	GAPDH	unclear	mouse	SMC-specific GAPDH overexpression→atherosclerosis↓			genetic *in vivo*	2020 [66]
restenosis	PGK1	glycolysis	rat	balloon-injured arteries	PGK1 protein ↑ (WB)	*In vitro*: si*PGK1*→ADP/ATP↑; AMPK↑ →VSMC proliferation, migration↓ *In vivo*: si*PGK1*→ neointima↓	knockdown *in vivo*	2021 [70]
atherosclerosis	PKM2	glycolysis	human, rabbit, mouse	OxLDL treated VSMCs; rabbit atherosclerotic plaques	PKM2 protein/activity↑; glucose uptake, ATP, ECAR↑	*In vitro*: PKM2 inhibitor→ECAR ↓→VSMC proliferation, migration↓ *In vivo*: PKM2 inhibitor→ atherosclerosis↓	Pharmacological *in vivo*	2019 [75]
restenosis	PKM2	glycolysis; non-glycolysis	human, mouse	carotid wire injury neointima	PKM2 protein↑	*In vitro*: *PKM2*-KO→ECAR↓→VSMC proliferation, migration↓ *In vivo*: SMC-*PKM2*-KO/PKM2 activator→neointima↓	genetic and pharmacological *in vivo*	2021 [72]
PAH	PKM2	glycolysis	rat, mouse	MCT-PAH rat lung tissue	PKM2, p-PKM2↑	*In vitro*: PKM2 inhibition→glucose consumption↓, lactate↓in mouse PASMC *In vivo*: PKM2 inhibition→PAH↓	correlative; pharmacological *in vivo*	2023 [106]
PAH	PKM2	glycolysis	rat	MCT-PAH rat lung tissue; serum	p-PKM2↑, pyruvate kinase activity↓; serum lactate↓	*In vitro*: rat PASMC→hypoxia→GPX1, GSH/GSSG↑ *In vivo*: PKM2 activators (L-serine and FBP→ PAH↓	correlative; pharmacological *in vivo*	2016 [107]
aneurysm	PKM2	glycolysis	human, mouse	scRNA-seq of aortic tissue from AD patients	VSMC glycolysis genes ↑	*In vivo*: PKM2 activator (TEPP–46)→ ECAR↑→ameurysm: no effect or the protective effect of GMRSP↓	correlative; pharmacological *in vivo*	2025 [148]
aneurysm	PKM2	glycolysis	human, mouse	aortic tissue from AD patients	serum lactate↑; histone lactylation↑	*In vitro*: PKM2 activator (TEPP–46)→ H3K18la↓, apoptosis↓ *In vivo*: TEPP–46→ ECAR↓→ameurysm↓	Pharmacological *in vivo*	2025 [145]
restenosis	LDHA	glycolysis; non-glycolysis	mouse; rat	proliferative VSMC; neointima	LDHA↑	*In vitro*: si*LDHA* → VSMC proliferation, migration↓ *In vivo*: ad*LDHA* OE→neointima↑	overexpression *in vivo*	2025 [79]
PAH	LDHA	glycolysis	mouse; rat	plasma and lung tissue of the PH mouse	LDHA↑; lactate↑	*In vitro*: si*LDHA*→ Akt↓→VSMC proliferation↓; lactate→VSMC proliferation↑ *In vivo*: AAV-sh*LDHA*/inhibitor→PAH↓	knockdown and pharmacological *in vivo*	2024 [111]
PAH	ENO1	glycolysis	human, mouse, rat	lung and PASMCs from IPAH patients; rat and mouse PAH lung	ENO1↑	*In vitro*: si*ENO1*/inhibitor→ECAR↓→VSMC proliferation, migration↓; apoptosis↑ *In vivo*: ENO1 inhibitor PAH↓	Pharmacological *in vivo*	2018 [103]
AD	ENO2	glycolysis	human, mouse	human aortic tissues	ENO2↑ (WB, RT-PCR, IHC)	SMC specific AAV-ENO2 knockdown→autophagy↓→AD↓	knockdown *in vivo*	2023 [152]
restenosis	G6PD	PPP	rat	aortas from rat with metabolic syndrome	G6PD activity↑; PPP metabolites↑	*In vitro*: si*G6PD*/G6PD inhibitor→SMC restricted genes↓; *In vivo*: G6PD inhibitor→neointima↓; G6PD^S188F^ variant rats→vascular resistance↓	genetic and pharmacological *in vivo*	2021 [82]
restenosis	G6PD	non-metabolic	mouse; rat	synthetic rat VSMCs	G6PD↑	*In vitro*: G6PD interacts with VDAC1→VSMC apoptosis↓ *In vivo*: ad*G6PD* OE→VSMC proliferation↑, apoptosis↓→neointima↑	overexpression *in vivo*	2025 [85]
Arterial stiffness	OGT	O-GlcNAc	mouse	constitutive αSMA-*Ogt* KO→contractile genes↓			genetic *in vivo*	2022 [92]
atherosclerosis	OGT	O-GlcNAc	Mouse	inducible SMC-*OGT*^KO^*ApoE*^*−/−*^ →contractile marker↑, inflammatory and proliferative marker↓→atherosclerosis↓			genetic *in vivo*	2023 [91]

**Abbreviations**: WB, western blot; IHC, Immunohistochemistry; OE, overexpression; AD, aortic dissection; KO, knockout.

**Table 2:** Summary of targeting glucose metabolism of VSMC in vascular disorders.

Target	Drug	Vascular disorder	Mechanism of action	specificity	Pharmacokinetics / Delivery	Off-target / Toxicity concerns	Translational readiness	Ref.
PFKFB3	3PO	PAH	Reduce collagen synthesis and proliferation of PASMCs	Low-moderate (inhibits PFKFB isoforms)	Poor bioavailability, short half-life	Broad metabolic effects	★★☆☆☆	[102]
PFKFB3	PFK15	Calcification	Inhibit osteogenic transdifferentiation of VSMCs	Improved selectivity vs 3PO	Limited PK data, IV delivery in preclinical models	Some cytotoxicity reported	★★☆☆☆	[128]
PKM2	SKN	Atherosclerosis	Inhibit proliferation and migration of VSMCs	Binds PKM2 but also affects ROS pathways	Poor oral bioavailability; unstable	Redox-related toxicity	★★☆☆☆	[75]
HK2	3-BrPA	PAH	Suppress PASMC proliferation and migration, enhance PASMC apoptosis	Non-specific alkylating agent	Rapid clearance, poor stability	High systemic toxicity	★☆☆☆☆	[99], [101]
G6PD	EPI	Neointimal formation	Regulate VSMC phenotypic fate	Moderate, androgen metabolite	Oral availability but low potency	Endocrine side effects	★★☆☆☆	[82]
G6PD	DHEA	PAH	Control VSMC phenotypic switch	Broad hormone precursor	Widely available supplement	Hormonal toxicity risks	★★☆☆☆	[114]
mTOR	PP242	PAH	Reduce proliferation and promote apoptosis of PASMCs	Potent ATP-competitive inhibitor	Well studied in oncology; oral bioavailability	Immunosuppression, metabolic effects	★★★★☆	[188]
HIF–1α	dominant-negative HIF–1α adenoviral construct	Neointimal formation	Reduce proliferation and promote apoptosis of VSMCs	Specific gene therapy	Viral delivery, poor systemic translation	Delivery/immune response risk	★☆☆☆☆	[183]

## Data Availability

No data was used for the research described in the article.

## References

[1] Owens GK, Regulation of differentiation of vascular smooth muscle cells, Physiol. Rev 75 (1995) 487–517, 10.1152/physrev.1995.75.3.487. 7624392

[2] Liu M, Gomez D, Smooth muscle cell phenotypic diversity, Arterioscler. Thromb. Vasc. Biol 39 (2019) 1715–1723, 10.1161/atvbaha.119.312131. 31340668 PMC6986347

[3] Kawai-Kowase K, Owens GK, Multiple repressor pathways contribute to phenotypic switching of vascular smooth muscle cells, Am. J. Physiol. Cell Physiol 292 (2007) C59–C69, 10.1152/ajpcell.00394.2006. 16956962

[4] Owens GK, , Molecular regulation of vascular smooth muscle cell differentiation in development and disease, Physiol. Rev 84 (2004) 767–801, 10.1152/physrev.00041.2003. 15269336

[5] Gomez D, Owens GK, Smooth muscle cell phenotypic switching in atherosclerosis, Cardiovasc Res 95 (2012) 156–164, 10.1093/cvr/cvs115. 22406749 PMC3388816

[6] Shankman LS, , KLF4-dependent phenotypic modulation of smooth muscle cells has a key role in atherosclerotic plaque pathogenesis, Nat. Med 21 (2015) 628–637, 10.1038/nm.3866. 25985364 PMC4552085

[7] Feil S, , Transdifferentiation of vascular smooth muscle cells to macrophage-like cells during atherogenesis, Circ. Res 115 (2014) 662–667, 10.1161/circresaha.115.304634. 25070003

[8] Clément M, , Vascular smooth muscle cell plasticity and autophagy in dissecting aortic aneurysms, Arterioscler. Thromb. Vasc. Biol 39 (2019) 1149–1159, 10.1161/ATVBAHA.118.311727. 30943775 PMC6544538

[9] Petsophonsakul P, , Role of vascular smooth muscle cell phenotypic switching and calcification in aortic aneurysm formation, Arterioscler. Thromb. Vasc. Biol 39 (2019) 1351–1368, 10.1161/atvbaha.119.312787. 31144989

[10] IYEMERE VP, , Vascular smooth muscle cell phenotypic plasticity and the regulation of vascular calcification, J. Intern. Med 260 (2006) 192–210, 10.1111/j.1365-2796.2006.01692.x. 16918817

[11] Stenmark KR, , Dynamic and diverse changes in the functional properties of vascular smooth muscle cells in pulmonary hypertension, Cardiovasc Res. 114 (2018) 551–564, 10.1093/cvr/cvy004. 29385432 PMC5852549

[12] Lechartier B, , Phenotypic diversity of vascular smooth muscle cells in pulmonary arterial hypertension: implications for therapy, CHEST 161 (2022) 219–231, 10.1016/j.chest.2021.08.040. 34391758

[13] Shi J, , Metabolism of vascular smooth muscle cells in vascular diseases, Am. J. Physiol. Heart Circ. Physiol 319 (2020) H613–h631, 10.1152/ajpheart.00220.2020. 32762559

[14] Eelen G, , Endothelial cell metabolism in normal and diseased vasculature, Circ. Res 116 (2015) 1231–1244, 10.1161/circresaha.116.302855. 25814684 PMC4380230

[15] Koelwyn GJ, , Regulation of macrophage immunometabolism in atherosclerosis, Nat. Immunol 19 (2018) 526–537, 10.1038/s41590-018-0113-3. 29777212 PMC6314674

[16] Sutendra G, , Fatty acid oxidation and malonyl-CoA decarboxylase in the vascular remodeling of pulmonary hypertension, Sci. Transl. Med 2 (2010) 44ra58, 10.1126/scitranslmed.3001327. 20702857

[17] Osman I, , TEAD1 (TEA Domain Transcription Factor 1) promotes smooth muscle cell proliferation through upregulating SLC1A5 (Solute Carrier Family 1 Member 5)-Mediated glutamine uptake, Circ. Res 124 (2019) 1309–1322, 10.1161/circresaha.118.314187. 30801233 PMC6493685

[18] Ma Q, , Purine synthesis suppression reduces the development and progression of pulmonary hypertension in rodent models, Eur. Heart J 44 (2023) 1265–1279, 10.1093/eurheartj/ehad044. 36721994 PMC10319969

[19] Ma Q, , ATIC-Associated de novo purine synthesis is critically involved in proliferative arterial disease, Circulation 146 (2022) 1444–1460, 10.1161/circulationaha.121.058901. 36073366 PMC9643655

[20] Adhikari N, , Increase in GLUT1 in smooth muscle alters vascular contractility and increases inflammation in response to vascular injury, Arterioscler. Thromb. Vasc. Biol 31 (2011) 86–94, 10.1161/ATVBAHA.110.215004. 20947823 PMC3014530

[21] Brand MD, Regulation analysis of energy metabolism, J. Exp. Biol 200 (1997) 193–202, 10.1242/jeb.200.2.193. 9050227

[22] Chen AN, , Lactylation, a novel metabolic reprogramming code: current status and prospects, Front. Immunol 12 (2021) 688910, 10.3389/fimmu.2021.688910. 34177945 PMC8222712

[23] Patra KC, Hay N, The pentose phosphate pathway and cancer, Trends Biochem Sci. 39 (2014) 347–354, 10.1016/j.tibs.2014.06.005. 25037503 PMC4329227

[24] Hart GW, , Cycling of O-linked beta-N-acetylglucosamine on nucleocytoplasmic proteins, Nature 446 (2007) 1017–1022, 10.1038/nature05815. 17460662

[25] Yang X, Qian K, Protein O-GlcNAcylation: emerging mechanisms and functions, Nat. Rev. Mol. Cell Biol 18 (2017) 452–465, 10.1038/nrm.2017.22. 28488703 PMC5667541

[26] Paul RJ, Functional compartmentalization of oxidative and glycolytic metabolism in vascular smooth muscle, Am. J. Physiol. Cell Physiol 244 (1983) C399–C409, 10.1152/ajpcell.1983.244.5.C399. 6846528

[27] Paul RJ, , Vascular smooth muscle: aerobic glycolysis linked to sodium and potassium transport processes, Science 206 (1979) 1414–1416, 10.1126/science.505014. 505014

[28] Suzuki LA, , Diabetes accelerates smooth muscle accumulation in lesions of atherosclerosis: lack of direct Growth-Promoting effects of high glucose levels, Diabetes 50 (2001) 851–860, 10.2337/diabetes.50.4.851. 11289052

[29] Paul RJ, Smooth muscle energetics, Annu. Rev. Physiol 51 (1989) 331–345, 10.1146/annurev.ph.51.030189.001555. 2653187

[30] Butler TM, Siegman MJ, High-Energy phosphate metabolism in vascular smooth muscle, Annu. Rev. Physiol 47 (1985) 629–643, 10.1146/annurev.ph.47.030185.003213. 3158271

[31] Lynch RM, Paul RJ, Compartmentation of glycolytic and glycogenolytic metabolism in vascular smooth muscle, Science 222 (1983) 1344–1346, 10.1126/science.6658455. 6658455

[32] Rabinowitz JD, Enerbäck S, Lactate: the ugly duckling of energy metabolism, Nat. Metab 2 (2020) 566–571, 10.1038/s42255-020-0243-4. 32694798 PMC7983055

[33] Shi J, , Metabolism of vascular smooth muscle cells in vascular diseases, Am. J. Physiol. Heart Circ. Physiol 319 (2020) H613–H631, 10.1152/ajpheart.00220.2020. 32762559

[34] Sun T, , The regulatory role and mechanism of energy metabolism in vascular diseases, FBL 29 (2024), 10.31083/j.fbl2901026. 38287818

[35] Clarkson TB, , Atherosclerosis in pigeons; its spontaneous occurrence and resemblance to human atherosclerosis, AMA Arch. Pathol 68 (1959) 143–147. 13669834

[36] Prichard RW, , Aortic atherosclerosis in pigeons and its complications, Arch. Pathol 77 (1964) 244–257. 14095741

[37] Zemplenyi T, Rosenstein AJ, Arterial enzymes and their relation to atherosclerosis in pigeons, Exp. Mol. Pathol 22 (1975) 225–241, 10.1016/0014-4800(75)90066-0. 163762

[38] Zemplényl T, Rosenstein AJ, Elevation of arterial phosphorfuctokinase activity associated with susceptibility to atherosclerosis in pigeons, Atherosclerosis 21 (1975) 167–177, 10.1016/0021-9150(75)90078-7. 236761

[39] Anderson JL, , Differentially expressed genes in aortic smooth muscle cells from atherosclerosis-susceptible and atherosclerosis-resistant pigeons, Poult. Sci 91 (2012) 1315–1325, 10.3382/ps.2011-01975. 22582288

[40] Rudd JH, , Imaging atherosclerotic plaque inflammation with [18F]-fluorodeoxyglucose positron emission tomography, Circulation 105 (2002) 2708–2711, 10.1161/01.cir.0000020548.60110.76. 12057982

[41] Pahk K, , Visualization of synthetic vascular smooth muscle cells in atherosclerotic carotid rat arteries by F-18 FDG PET, Sci. Rep 7 (2017) 6989, 10.1038/s41598-017-07073-3. 28765576 PMC5539104

[42] Tomas L, , Altered metabolism distinguishes high-risk from stable carotid atherosclerotic plaques, Eur. Heart J 39 (2018) 2301–2310, 10.1093/eurheartj/ehy124. 29562241 PMC6012762

[43] Seeley EH, , Spatially resolved metabolites in stable and unstable human atherosclerotic plaques identified by mass spectrometry imaging, Arterioscler. Thromb. Vasc. Biol 43 (2023) 1626–1635, 10.1161/atvbaha.122.318684. 37381983 PMC10527524

[44] Guillermier C, , Imaging mass spectrometry reveals heterogeneity of proliferation and metabolism in atherosclerosis, JCI Insight 4 (2019), 10.1172/jci.insight.128528. PMC6629145 31167964

[45] Kaiser N, , Differential regulation of glucose transport and transporters by glucose in vascular endothelial and smooth muscle cells, Diabetes 42 (1993) 80–89, 10.2337/diab.42.1.80. 7678404

[46] Pyla R, , Expression of conventional and novel glucose transporters, GLUT1, −9, −10, and −12, in vascular smooth muscle cells, Am. J. Physiol. Cell Physiol 304 (2013) C574–C589, 10.1152/ajpcell.00275.2012. 23302780 PMC3671567

[47] Shepherd PR, Kahn BB, Glucose transporters and insulin action–implications for insulin resistance and diabetes mellitus, N. Engl. J. Med 341 (1999) 248–257, 10.1056/nejm199907223410406. 10413738

[48] Shepherd PR, , Phosphoinositide 3-kinase: the key switch mechanism in insulin signalling, Biochem J. 333 (Pt 3) (1998) 471–490, 10.1042/bj3330471. 9677303 PMC1219607

[49] Hall JL, , Upregulation of glucose metabolism during intimal lesion formation is coupled to the inhibition of vascular smooth muscle cell apoptosis: role of GSK3β, Diabetes 50 (2001) 1171–1179, 10.2337/diabetes.50.5.1171. 11334423

[50] Wall VZ, , Smooth muscle glucose metabolism promotes monocyte recruitment and atherosclerosis in a mouse model of metabolic syndrome, JCI Insight 3 (2018), 10.1172/jci.insight.96544. PMC6124428 29875324

[51] Docherty CK, , Impaired mitochondrial respiration in human carotid plaque atherosclerosis: a potential role for Pink1 in vascular smooth muscle cell energetics, Atherosclerosis 268 (2018) 1–11, 10.1016/j.atherosclerosis.2017.11.009. 29156421 PMC6565844

[52] Ni Z, , Recipient c-Kit lineage cells repopulate smooth muscle cells of transplant arteriosclerosis in mouse models, Circ. Res 125 (2019) 223–241, 10.1161/CIRCRESAHA.119.314855. 31079549 PMC6615935

[53] Heiss EH, , Increased aerobic glycolysis is important for the motility of activated VSMC and inhibited by indirubin-3′-monoxime, Vasc. Pharm 83 (2016) 47–56, 10.1016/j.vph.2016.05.002. PMC4939873 27185663

[54] Tang Y, , MFN2 prevents neointimal hyperplasia in vein grafts via destabilizing PFK1, Circ. Res 130 (2022) e26–e43, 10.1161/circresaha.122.320846. 35450439

[55] Rider MH, , 6-phosphofructo-2-kinase/fructose-2,6-bisphosphatase: head-to-head with a bifunctional enzyme that controls glycolysis, Biochem J. 381 (2004) 561–579, 10.1042/bj20040752. 15170386 PMC1133864

[56] Pilkis SJ, , 6-Phosphofructo-2-kinase/fructose-2,6-bisphosphatase: a metabolic signaling enzyme, Annu Rev. Biochem 64 (1995) 799–835, 10.1146/annurev.bi.64.070195.004055. 7574501

[57] Okar DA, , Regulation of the regulatory enzyme, 6-phosphofructo-2-kinase/fructose-2,6-bisphosphatase, Adv. Enzym. Regul 44 (2004) 123–154, 10.1016/j.advenzreg.2003.11.006. 15581487

[58] Okar DA, , PFK-2/FBPase-2: maker and breaker of the essential biofactor fructose-2,6-bisphosphate, Trends Biochem Sci. 26 (2001) 30–35. 11165514 10.1016/s0968-0004(00)01699-6

[59] Poels K, , Inhibition of PFKFB3 hampers the progression of atherosclerosis and promotes plaque stability, Front Cell Dev. Biol 8 (2020) 581641, 10.3389/fcell.2020.581641. PMC7688893 33282864

[60] Tillie RJHA, , Partial inhibition of the 6-Phosphofructo-2-Kinase/Fructose-2,6-Bisphosphatase-3 (PFKFB3) enzyme in myeloid cells does not affect atherosclerosis, Front. Cell Dev. Biol 9 (2021), 10.3389/fcell.2021.695684. PMC8387953 34458258

[61] Guo S, , Gene-dosage effect of Pfkfb3 on monocyte/macrophage biology in atherosclerosis, Br. J. Pharm 179 (2022) 4974–4991, 10.1111/bph.15926. PMC10420406 35834356

[62] Perrotta P, , PFKFB3 gene deletion in endothelial cells inhibits intraplaque angiogenesis and lesion formation in a murine model of venous bypass grafting, Angiogenesis 25 (2022) 129–143, 10.1007/s10456-021-09816-3. 34432198 PMC8813728

[63] Cao K, , Glycolysis and de novo fatty acid synthesis cooperatively regulate pathological vascular smooth muscle cell phenotypic switching and neointimal hyperplasia, J. Pathol 259 (2023) 388–401, 10.1002/path.6052. 36640260

[64] Zhang X, , KLF4-PFKFB3-driven glycolysis is essential for phenotypic switching of vascular smooth muscle cells, Commun. Biol 5 (2022) 1332, 10.1038/s42003-022-04302-y. 36470917 PMC9722670

[65] Sukhanov S, , Novel effect of oxidized Low-Density lipoprotein, Circ. Res 99 (2006) 191–200, 10.1161/01.RES.0000232319.02303.8c. 16778134

[66] Sukhanov S, , Atheroprotective effects induced by Glyceraldehyde-3′-phosphate dehydrogenase (GAPDH) in murine model of atherosclerosis, −1, FASEB J. 34 (2020) 1, 10.1096/fasebj.2020.34.s1.03693.

[67] Perrotta I, , Expression profile and subcellular localization of GAPDH in the smooth muscle cells of human atherosclerotic plaque: an immunohistochemical and ultrastructural study with biological therapeutic perspectives, Microsc. Micro 20 (2014) 1145–1157, 10.1017/s1431927614001020. 24851941

[68] Nicholls C, , GAPDH: a common enzyme with uncommon functions, Clin. Exp. Pharm. Physiol 39 (2012) 674–679, 10.1111/j.1440-1681.2011.05599.x. 21895736

[69] Hou X, , Nuclear complex of glyceraldehyde-3-phosphate dehydrogenase and DNA repair enzyme apurinic/apyrimidinic endonuclease I protect smooth muscle cells against oxidant-induced cell death, Faseb J. 31 (2017) 3179–3192, 10.1096/fj.201601082R. 28404743 PMC5471514

[70] Pan CH, , Pathological role of phosphoglycerate kinase 1 in balloon Angioplasty-Induced neointima formation, Int J. Mol. Sci 22 (2021), 10.3390/ijms22168822. PMC8396187 34445528

[71] He P, , PKM2 is a key factor to regulate neurogenesis and cognition by controlling lactate homeostasis, Stem Cell Rep. 20 (2025) 102381, 10.1016/j.stemcr.2024.11.011. PMC11784464 39706177

[72] Jain M, , Smooth muscle Cell–Specific PKM2 (Pyruvate Kinase Muscle 2) promotes smooth muscle cell phenotypic switching and neointimal hyperplasia, Arterioscler. Thromb. Vasc. Biol 41 (2021) 1724–1737, 10.1161/ATVBAHA.121.316021. 33691477 PMC8062279

[73] Toller-Kawahisa JE, , The metabolic function of pyruvate kinase M2 regulates reactive oxygen species production and microbial killing by neutrophils, Nat. Commun 14 (2023) 4280, 10.1038/s41467-023-40021-6. 37460614 PMC10352279

[74] Liang LJ, , CIP2A induces PKM2 tetramer formation and oxidative phosphorylation in non-small cell lung cancer, Cell Discov. 10 (2024) 13, 10.1038/s41421-023-00633-0. 38321019 PMC10847417

[75] Zhao X, , PKM2-dependent glycolysis promotes the proliferation and migration of vascular smooth muscle cells during atherosclerosis, Acta Biochim. Et. Biophys. Sin 52 (2019) 9–17, 10.1093/abbs/gmz135. 31867609

[76] Jia Y, , PHB2 maintains the contractile phenotype of VSMCs by counteracting PKM2 splicing, Circ. Res 131 (2022) 807–824, 10.1161/circresaha.122.321005. 36200440

[77] Cai Z, , Regulation of Ptbp1-controlled alternative splicing of pyruvate kinase muscle by liver kinase b1 governs vascular smooth muscle cell plasticity in vivo, Cardiovasc Res (2024), 10.1093/cvr/cvae187. PMC11587553 39189621

[78] Kim JH, , Lactate dehydrogenase-A is indispensable for vascular smooth muscle cell proliferation and migration, Biochem Biophys. Res Commun 492 (2017) 41–47, 10.1016/j.bbrc.2017.08.041. 28818664

[79] Chen ZH, , Lactate dehydrogenase a crotonylation and Mono-Ubiquitination maintains vascular smooth muscle cell growth and migration and promotes neointima hyperplasia, J. Am. Heart Assoc 14 (2025) e036377, 10.1161/jaha.124.036377. 40028887 PMC12132763

[80] TeSlaa T, , The pentose phosphate pathway in health and disease, Nat. Metab 5 (2023) 1275–1289, 10.1038/s42255-023-00863-2. 37612403 PMC11251397

[81] Dong LH, , TRAF6-Mediated SM22α K21 ubiquitination promotes G6PD activation and NADPH production, contributing to GSH homeostasis and VSMC survival in vitro and in vivo, Circ. Res 117 (2015) 684–694, 10.1161/circresaha.115.306233. 26291555

[82] Dhagia V, , G6PD activity contributes to the regulation of histone acetylation and gene expression in smooth muscle cells and to the pathogenesis of vascular diseases, Am. J. Physiol. Heart Circ. Physiol 320 (2021) H999–h1016, 10.1152/ajpheart.00488.2020. 33416454 PMC7988761

[83] Koval OM, , Mitochondrial calcium uniporter regulates metabolic remodeling and smooth muscle cell proliferation in type 2 diabetes, J. Am. Heart Assoc 14 (2025) e039220, 10.1161/JAHA.124.039220. 40673552 PMC12449995

[84] Ata H, , Mechanism of glucose-6-phosphate dehydrogenase-mediated regulation of coronary artery contractility, Am. J. Physiol. Heart Circ. Physiol 300 (2011) H2054–H2063, 10.1152/ajpheart.01155.2010. 21398595 PMC3119095

[85] Zhang T, , G6PD maintains the VSMC synthetic phenotype and accelerates vascular neointimal hyperplasia by inhibiting the VDAC1–Bax-mediated mitochondrial apoptosis pathway, Cell. Mol. Biol. Lett 29 (2024) 47, 10.1186/s11658-024-00566-w. 38589823 PMC11003121

[86] Chettimada S, , Vascular smooth muscle cell contractile protein expression is increased through protein kinase G-dependent and -independent pathways by glucose-6-phosphate dehydrogenase inhibition and deficiency, Am. J. Physiol. Heart Circ. Physiol 311 (2016) H904–h912, 10.1152/ajpheart.00335.2016. 27521420 PMC5114469

[87] Federici M, , Insulin-dependent activation of endothelial nitric oxide synthase is impaired by O-linked glycosylation modification of signaling proteins in human coronary endothelial cells, Circulation 106 (2002) 466–472, 10.1161/01.cir.0000023043.02648.51. 12135947

[88] Zhang H, , Glutamine supplementation alleviated aortic atherosclerosis in mice model and in vitro, Proteomics 24 (2024) e2300179, 10.1002/pmic.202300179. 37679095

[89] Raman P, , Glycosylation mediates up-regulation of a potent antiangiogenic and proatherogenic protein, thrombospondin-1, by glucose in vascular smooth muscle cells, J. Biol. Chem 282 (2007) 5704–5714, 10.1074/jbc.M610965200. 17178709

[90] Ganguly R, , Trivalent chromium inhibits TSP-1 expression, proliferation, and O-GlcNAc signaling in vascular smooth muscle cells in response to high glucose in vitro, Am. J. Physiol. Cell Physiol 308 (2015) C111–C122, 10.1152/ajpcell.00256.2014. 25354527

[91] Khanal S, , Deletion of smooth muscle O-GlcNAc transferase prevents development of atherosclerosis in western diet-fed hyperglycemic ApoE(−/−) mice in vivo, Int J. Mol. Sci 24 (2023), 10.3390/ijms24097899. PMC10178779 37175604

[92] Xiong X, , αSMA-Cre-mediated ogt deletion leads to heart failure and vascular smooth muscle cell dysfunction in mice, Biochem Biophys. Res Commun 625 (2022) 31–37, 10.1016/j.bbrc.2022.07.106. 35944361

[93] Lima VV, , O-GlcNAcylation contributes to the vascular effects of ET-1 via activation of the RhoA/Rho-kinase pathway, Cardiovasc Res 89 (2011) 614–622, 10.1093/cvr/cvq338. 20978008 PMC3028974

[94] Bolanle IO, , Revascularisation of type 2 diabetics with coronary artery disease: insights and therapeutic targeting of O-GlcNAcylation, Nutr. Metab. Cardiovasc Dis 31 (2021) 1349–1356, 10.1016/j.numecd.2021.01.017. 33812732

[95] Marsboom G, , Lung 18F-fluorodeoxyglucose positron emission tomography for diagnosis and monitoring of pulmonary arterial hypertension, Am. J. Respir. Crit. Care Med 185 (2012) 670–679, 10.1164/rccm.201108-1562OC. 22246173 PMC3326289

[96] Xiao Y, , PDGF promotes the warburg effect in pulmonary arterial smooth muscle cells via activation of the PI3K/AKT/mTOR/HIF-1α signaling pathway, Cell. Physiol. Biochem 42 (2017) 1603–1613, 10.1159/000479401. 28738389

[97] Loberg RD, , Enhanced glycogen synthase kinase-3beta activity mediates hypoxia-induced apoptosis of vascular smooth muscle cells and is prevented by glucose transport and metabolism, J. Biol. Chem 277 (2002) 41667–41673, 10.1074/jbc.M206405200. 12200436

[98] Lin Z, , GLUT-1 reduces hypoxia-induced apoptosis and JNK pathway activation, Am. J. Physiol. Endocrinol. Metab 278 (2000) E958–E966, 10.1152/ajpendo.2000.278.5.E958. 10780954

[99] Chen F, , 3-Bromopyruvate reverses hypoxia-induced pulmonary arterial hypertension through inhibiting glycolysis: in vitro and in vivo studies, Int J. Cardiol 266 (2018) 236–241, 10.1016/j.ijcard.2018.03.104. 29735421

[100] Luo L, , miR-125a-5p inhibits glycolysis by targeting hexokinase-II to improve pulmonary arterial hypertension, Aging (Albany NY) 12 (2020) 9014–9030, 10.18632/aging.103163. 32427576 PMC7288947

[101] Zhang YL, , 3-Bromopyruvate attenuates experimental pulmonary hypertension via inhibition of glycolysis, Am. J. Hypertens 32 (2019) 426–432, 10.1093/ajh/hpy191. 30561502

[102] Kovacs L, , PFKFB3 in smooth muscle promotes vascular remodeling in pulmonary arterial hypertension, Am. J. Respir. Crit. Care Med 200 (2019) 617–627, 10.1164/rccm.201812-2290OC. 30817168 PMC6727156

[103] Dai J, , Alpha-enolase regulates the malignant phenotype of pulmonary artery smooth muscle cells via the AMPK-Akt pathway, Nat. Commun 9 (2018) 3850, 10.1038/s41467-018-06376-x. 30242159 PMC6155017

[104] Kato Y, , Anti-enolase 1 antibodies from a patient with systemic lupus erythematosus accompanied by pulmonary arterial hypertension promote migration of pulmonary artery smooth muscle cells, Immunol. Lett 218 (2020) 22–29, 10.1016/j.imlet.2019.12.005. 31866401

[105] Bhedi CD, , Glycolysis regulated transglutaminase 2 activation in cardiopulmonary fibrogenic remodeling, Faseb J. 34 (2020) 930–944, 10.1096/fj.201902155R. 31914588 PMC6956703

[106] Li W, , Shikonin improves pulmonary vascular remodeling in monocrotaline-induced pulmonary arterial hypertension via regulation of PKM2, Mol. Med Rep 27 (2023), 10.3892/mmr.2023.12947. PMC9936259 36734266

[107] Guo D, , Inhibition of pyruvate kinase M2 by reactive oxygen species contributes to the development of pulmonary arterial hypertension, J. Mol. Cell Cardiol 91 (2016) 179–187, 10.1016/j.yjmcc.2016.01.009. 26774701

[108] Zhang A, , Pyruvate kinase M2 activation protects against the proliferation and migration of pulmonary artery smooth muscle cells, Cell Tissue Res 382 (2020) 585–598, 10.1007/s00441-020-03245-2. 32719938

[109] Takubo K, , Regulation of glycolysis by pdk functions as a metabolic checkpoint for cell cycle quiescence in hematopoietic stem cells, Cell Stem Cell 12 (2013) 49–61, 10.1016/j.stem.2012.10.011. 23290136 PMC6592822

[110] Li M, , SIRT6 inhibits hypoxia-induced pulmonary arterial smooth muscle cells proliferation via HIF-1α/PDK4 signaling, Life Sci. 312 (2023) 121192, 10.1016/j.lfs.2022.121192. 36396113

[111] Wu D, , Lactate dehydrogenase a (LDHA)-mediated lactate generation promotes pulmonary vascular remodeling in pulmonary hypertension, J. Transl. Med 22 (2024) 738, 10.1186/s12967-024-05543-7. 39103838 PMC11302077

[112] Zhao Y, , Metabolomic heterogeneity of pulmonary arterial hypertension, PLOS ONE 9 (2014) e88727, 10.1371/journal.pone.0088727. 24533144 PMC3923046

[113] Boehme J, , Pulmonary artery smooth muscle cell hyperproliferation and metabolic shift triggered by pulmonary overcirculation, Am. J. Physiol. Heart Circ. Physiol 311 (2016) H944–H957, 10.1152/ajpheart.00040.2016. 27591215 PMC5114466

[114] Chettimada S, , Hypoxia-induced glucose-6-phosphate dehydrogenase overexpression and -activation in pulmonary artery smooth muscle cells: implication in pulmonary hypertension, Am. J. Physiol. Lung Cell Mol. Physiol 308 (2015) L287–L300, 10.1152/ajplung.00229.2014. 25480333 PMC4338932

[115] Chettimada S, , Glc-6-PD and PKG contribute to hypoxia-induced decrease in smooth muscle cell contractile phenotype proteins in pulmonary artery, Am. J. Physiol. Lung Cell Mol. Physiol 303 (2012) L64–L74, 10.1152/ajplung.00002.2012. 22582112 PMC3426433

[116] Lakhkar A, , 20-HETE-induced mitochondrial superoxide production and inflammatory phenotype in vascular smooth muscle is prevented by glucose-6-phosphate dehydrogenase inhibition, Am. J. Physiol. Heart Circ. Physiol 310 (2016) H1107–H1117, 10.1152/ajpheart.00961.2015. 26921441 PMC4867393

[117] Barnes JW, , O-GlcNAc transferase regulates angiogenesis in idiopathic pulmonary arterial hypertension, Int J. Mol. Sci 20 (2019), 10.3390/ijms20246299. PMC6941156 31847126

[118] Prisco SZ, , Excess protein O-GlcNAcylation links metabolic derangements to right ventricular dysfunction in pulmonary arterial hypertension, Int J. Mol. Sci 21 (2020), 10.3390/ijms21197278. PMC7582480 33019763

[119] Prisco SZ, , With no lysine kinase 1 promotes metabolic derangements and RV dysfunction in pulmonary arterial hypertension, JACC Basic Transl. Sci 6 (2021) 834–850, 10.1016/j.jacbts.2021.09.004. 34869947 PMC8617575

[120] Aulak KS, , Specific O-GlcNAc modification at Ser-615 modulates eNOS function, Redox Biol. 36 (2020) 101625, 10.1016/j.redox.2020.101625. 32863226 PMC7334407

[121] Barnes JW, , O-linked β-N-acetylglucosamine transferase directs cell proliferation in idiopathic pulmonary arterial hypertension, Circulation 131 (2015) 1260–1268, 10.1161/circulationaha.114.013878. 25663381 PMC4390469

[122] Rufino M, , The GLUT-1 xbaI gene polymorphism is associated with vascular calcifications in nondiabetic uremic patients, Nephron Clin. Pr 108 (2008) c182–c187, 10.1159/000118940. 18311082

[123] Zhu Y, , Periostin promotes arterial calcification through PPARγ-related glucose metabolism reprogramming, Am. J. Physiol. Heart Circ. Physiol 320 (2021) H2222–h2239, 10.1152/ajpheart.01009.2020. 33834866

[124] Tzoulaki I, , Serum metabolic signatures of coronary and carotid atherosclerosis and subsequent cardiovascular disease, Eur. Heart J 40 (2019) 2883–2896, 10.1093/eurheartj/ehz235. 31102408 PMC7963131

[125] Idelevich A, , Bone gla protein increases HIF-1alpha-dependent glucose metabolism and induces cartilage and vascular calcification, Arterioscler. Thromb. Vasc. Biol 31 (2011) e55–e71, 10.1161/atvbaha.111.230904. 21757657

[126] Rashdan NA, , Osteocalcin regulates arterial calcification via altered wnt signaling and glucose metabolism, J. Bone Miner. Res 35 (2019) 357–367, 10.1002/jbmr.3888. 31596966

[127] Niu J, , κ-opioid receptor stimulation alleviates rat vascular smooth muscle cell calcification via PFKFB3-lactate signaling, Aging (Albany NY) 13 (2021) 14355–14371, 10.18632/aging.203050. 34016793 PMC8202865

[128] Chen J, , PFKFB3-driven vascular smooth muscle cell glycolysis promotes vascular calcification via the altered FoxO3 and lactate production, FASEB J. 37 (2023) e23182, 10.1096/fj.202300900R. 37682013

[129] Lee SJ, , Pyruvate dehydrogenase kinase 4 promotes vascular calcification via SMAD1/5/8 phosphorylation, Sci. Rep 5 (2015) 16577, 10.1038/srep16577. 26560812 PMC4642318

[130] Ma W-Q, , PDK4 promotes vascular calcification by interfering with autophagic activity and metabolic reprogramming, Cell Death Dis. 11 (2020) 991, 10.1038/s41419-020-03162-w. 33203874 PMC7673024

[131] Zhu Y, , Advanced glycation end products accelerate calcification in VSMCs through HIF-1α/PDK4 activation and suppress glucose metabolism, Sci. Rep 8 (2018) 13730, 10.1038/s41598-018-31877-6. 30213959 PMC6137084

[132] Carr JJ, , Calcified atherosclerotic plaque and bone mineral density in type 2 diabetes: the diabetes heart study, Bone 42 (2008) 43–52, 10.1016/j.bone.2007.08.023. 17964237 PMC2239236

[133] Stabley JN, Towler DA, Arterial calcification in diabetes mellitus: preclinical models and translational implications, Arterioscler. Thromb. Vasc. Biol 37 (2017) 205–217, 10.1161/atvbaha.116.306258. 28062508 PMC5480317

[134] Heath JM, , Activation of AKT by O-linked N-acetylglucosamine induces vascular calcification in diabetes mellitus, Circ. Res 114 (2014) 1094–1102, 10.1161/circresaha.114.302968. 24526702 PMC4030422

[135] Zhang W, , Impaired intracellular calcium homeostasis enhances protein O-GlcNAcylation and promotes vascular calcification and stiffness in diabetes, Redox Biol. 63 (2023) 102720, 10.1016/j.redox.2023.102720. 37230005 PMC10225928

[136] Bossone E, Eagle KA, Epidemiology and management of aortic disease: aortic aneurysms and acute aortic syndromes, Nat. Rev. Cardiol 18 (2021) 331–348, 10.1038/s41569-020-00472-6. 33353985

[137] Lu H, , Vascular smooth muscle cells in aortic aneurysm: from genetics to mechanisms, J. Am. Heart Assoc 10 (2021) e023601, 10.1161/jaha.121.023601. 34796717 PMC9075263

[138] Lederle FA, , Prevalence and associations of abdominal aortic aneurysm detected through screening. Aneurysm Detection and Management (ADAM) Veterans Affairs Cooperative Study Group, Ann. Intern Med 126 (1997) 441–449, 10.7326/0003-4819-126-6-199703150-00004. 9072929

[139] Lederle FA, , The aneurysm detection and management study screening program: validation cohort and final results. Aneurysm Detection and Management Veterans Affairs Cooperative Study Investigators, Arch. Intern Med 160 (2000) 1425–1430, 10.1001/archinte.160.10.1425. 10826454

[140] Morris DR, , Opposite associations of aortic aneurysm with blood glucose and with diabetes mellitus, Circulation 140 (2019) 264–266, 10.1161/CIRCULATIONAHA.119.040398. 31306072

[141] Li Y, , Treatment with small molecule inhibitors of advanced glycation end-products formation and advanced glycation end-products-mediated collagen Cross-Linking promotes experimental aortic aneurysm progression in diabetic mice, J. Am. Heart Assoc 12 (2023) e028081, 10.1161/JAHA.122.028081. 37158066 PMC10227285

[142] Raffort J, , Diabetes and aortic aneurysm: current state of the art, Cardiovasc Res 114 (2018) 1702–1713, 10.1093/cvr/cvy174. 30052821 PMC6198737

[143] Dattani N, , Diabetes mellitus and abdominal aortic aneurysms: a review of the mechanisms underlying the negative relationship, Diab Vasc. Dis. Res 15 (2018) 367–374, 10.1177/1479164118780799. 29874945

[144] Shanmuganathan G, Agrawal DK, Diabetes and abdominal aortic aneurysm: is the protective effect on AAA due to antidiabetic medications alone, due to the disease alone, or both? Arch. Intern Med Res 7 104113 (2024) 10.26502/aimr.0169. PMC11156236 38846325

[145] Li YE, , March2 alleviates aortic Aneurysm/Dissection by regulating PKM2 polymerization, Circ. Res 136 (2025) e73–e93, 10.1161/circresaha.124.325049. 40079144

[146] Kotze CW, , Increased metabolic activity in abdominal aortic aneurysm detected by 18F-fluorodeoxyglucose (18F-FDG) positron emission tomography/computed tomography (PET/CT), Eur. J. Vasc. Endovasc. Surg 38 (2009) 93–99, 10.1016/j.ejvs.2008.12.016. 19217326

[147] Tsuruda T, , Inhibition of development of abdominal aortic aneurysm by glycolysis restriction, Arterioscler. Thromb. Vasc. Biol 32 (2012) 1410–1417, 10.1161/atvbaha.111.237065. 22499992

[148] Wang J, , GMRSP encoded by lncRNA H19 regulates metabolic reprogramming and alleviates aortic dissection, Nat. Commun 16 (2025) 1719, 10.1038/s41467-025-57011-5. 39966416 PMC11836370

[149] Low BC, , Angiotensin II stimulates glucose transport activity in cultured vascular smooth muscle cells, J. Biol. Chem 267 (1992) 20740–20745, 10.1016/S0021-9258(19)36748-1. 1400389

[150] Torimoto K, , Glucose consumption of vascular cell types in culture: toward optimization of experimental conditions, Am. J. Physiol. Cell Physiol 322 (2022) C73–C85, 10.1152/ajpcell.00257.2021. 34817269 PMC8791793

[151] Torimoto K, , Glucose transporter 1 in vascular smooth muscle cells is dispensable for abdominal aortic aneurysm induced by angiotensin II, JVS Vasc. Sci 6 (2025) 100270, 10.1016/j.jvssci.2024.100270. 39811041 PMC11728061

[152] Cai W, , Elevated ENO2 disrupts VSMCs homeostasis facilitating the development of aortic dissection, 2003.2023.534041. bioRxiv 2023 (2023) 10.1101/2023.03.23.534041.

[153] Oller J, , Extracellular tuning of mitochondrial respiration leads to aortic aneurysm, Circulation 143 (2021) 2091–2109, 10.1161/circulationaha.120.051171. 33709773 PMC8140666

[154] Deng Y, , Abstract 4146210: TCF7L2 deficiency in vascular smooth muscle cells mitigates abdominal aortic aneurysm, -A4146210, Circulation 150 (2024) A4146210, 10.1161/circ.150.suppl_1.4146210.

[155] Anastasiou D, , Pyruvate kinase M2 activators promote tetramer formation and suppress tumorigenesis, Nat. Chem. Biol 8 (2012) 839–847, 10.1038/nchembio.1060. 22922757 PMC3711671

[156] Angiari S, , Pharmacological activation of pyruvate kinase M2 inhibits CD4(+) t cell pathogenicity and suppresses autoimmunity, e398, Cell Metab. 31 (2020) 391–405, 10.1016/j.cmet.2019.10.015. 31761564 PMC7001035

[157] Wiese EK, , Enzymatic activation of pyruvate kinase increases cytosolic oxaloacetate to inhibit the warburg effect, Nat. Metab 3 (2021) 954–968, 10.1038/s42255-021-00424-5. 34226744 PMC8316326

[158] Magistretti PJ, Allaman I, Lactate in the brain: from metabolic end-product to signalling molecule, Nat. Rev. Neurosci 19 (2018) 235–249, 10.1038/nrn.2018.19. 29515192

[159] Faubert B, , Lactate metabolism in human lung tumors, e359, Cell 171 (2017) 358–371, 10.1016/j.cell.2017.09.019. 28985563 PMC5684706

[160] Sonveaux P, , Targeting lactate-fueled respiration selectively kills hypoxic tumor cells in mice, J. Clin. Invest 118 39303942 (2008), 10.1172/JCI36843. PMC2582933 19033663

[161] Zhang H, , Lactate metabolism and lactylation in cardiovascular disease: novel mechanisms and therapeutic targets, Front Cardiovasc Med. 11 (2024) 1489438, 10.3389/fcvm.2024.1489438. 39664763 PMC11631895

[162] Zhu S, , Inactivation of monocarboxylate transporter MCT3 by DNA methylation in atherosclerosis, Circulation 112 (2005) 1353–1361, 10.1161/circulationaha.104.519025. 16116050

[163] Yang L, , Lactate promotes synthetic phenotype in vascular smooth muscle cells, Circ. Res 121 (2017) 1251–1262, 10.1161/CIRCRESAHA.117.311819. 29021296 PMC5681426

[164] Zhu Y, , Lactate accelerates vascular calcification through NR4A1-regulated mitochondrial fission and BNIP3-related mitophagy, Apoptosis 25 (2020) 321–340, 10.1007/s10495-020-01592-7. 31993850

[165] Zhu Y, , Lactate accelerates calcification in VSMCs through suppression of BNIP3-mediated mitophagy, Cell Signal 58 (2019) 53–64, 10.1016/j.cellsig.2019.03.006. 30851408

[166] Zhu Y, , Exploring a new mechanism between lactate and VSMC calcification: PARP1/POLG/UCP2 signaling pathway and imbalance of mitochondrial homeostasis, Cell Death Dis. 14 (2023) 598, 10.1038/s41419-023-06113-3. 37679327 PMC10484939

[167] Zhang D, , Metabolic regulation of gene expression by histone lactylation, Nature 574 (2019) 575–580, 10.1038/s41586-019-1678-1. 31645732 PMC6818755

[168] Niu Z, , HBO1 catalyzes lysine lactylation and mediates histone H3K9la to regulate gene transcription, Nat. Commun 15 (2024) 3561, 10.1038/s41467-024-47900-6. 38670996 PMC11053077

[169] Gonzatti MB, , Class I histone deacetylases catalyze lysine lactylation, J. Biol. Chem 301 (2025) 110602, 10.1016/j.jbc.2025.110602. 40835008 PMC12624779

[170] Gaffney DO, , Non-enzymatic lysine lactoylation of glycolytic enzymes, e206, Cell Chem. Biol 27 (2020) 206–213, 10.1016/j.chembiol.2019.11.005. 31767537 PMC7395678

[171] Xu X, , Sox10 escalates vascular inflammation by mediating vascular smooth muscle cell transdifferentiation and pyroptosis in neointimal hyperplasia, Cell Rep. 42 (2023) 112869, 10.1016/j.celrep.2023.112869. 37481722

[172] Li X, , TRAP1 drives smooth muscle cell senescence and promotes atherosclerosis via HDAC3-primed histone H4 lysine 12 lactylation, Eur. Heart J (2024), 10.1093/eurheartj/ehae379. PMC11481199 39088352

[173] Ma W, , Orphan nuclear receptor NR4A3 promotes vascular calcification via histone lactylation, Circ. Res 134 (2024) 1427–1447, 10.1161/circresaha.123.323699. 38629274

[174] Zhu J.Z. Yi, Wang Fangfang, Liu Rui-ping, H3K18 lactylation-mediated, CHI3L1 Expr. Exacerbates Diabet. Arter. Calcif (2024). 〈https://papers.ssrn.com/sol3/papers.cfm?abstract_id=4831857〉.

[175] Chen J, , Histone lactylation driven by mROS-mediated glycolytic shift promotes hypoxic pulmonary hypertension, J. Mol. Cell Biol 14 (2023), 10.1093/jmcb/mjac073. PMC10175659 36564027

[176] Zhu X, , Super Enhancer-driven LncRNA UNC5B-AS1 inhibits inflammatory phenotypic transition in smooth muscle cells via lactylation modification, 2005.2007.593065. bioRxiv 2024 (2024) 10.1101/2024.05.07.593065. 40336475

[177] Zhu W, , Lactate and lactylation in cardiovascular diseases: current progress and future perspectives, Metabolism 158 (2024) 155957, 10.1016/j.metabol.2024.155957. 38908508

[178] Semenza GL, HIF-1: mediator of physiological and pathophysiological responses to hypoxia, J. Appl. Physiol 88 (2000) 1474–1480, 10.1152/jappl.2000.88.4.1474. 10749844

[179] Mathupala SP, , Glucose catabolism in cancer cells: identification and characterization of a marked activation response of the type II Hexokinase gene to hypoxic conditions *, J. Biol. Chem 276 (2001) 43407–43412, 10.1074/jbc.M108181200. 11557773

[180] w Kim J, , HIF-1-mediated expression of pyruvate dehydrogenase kinase: a metabolic switch required for cellular adaptation to hypoxia, Cell Metab. 3 (2006) 177–185, 10.1016/j.cmet.2006.02.002. 16517405

[181] Semenza GL, Hypoxia-inducible factor 1 and cardiovascular disease, Annu Rev. Physiol 76 (2014) 39–56, 10.1146/annurev-physiol-021113-170322. 23988176 PMC4696033

[182] Sousa Fialho MDL, , Hypoxia-inducible factor 1 signalling, metabolism and its therapeutic potential in cardiovascular disease, Biochim. Biophys. Acta Mol. Basis Dis 1865 (2019) 831–843, 10.1016/j.bbadis.2018.09.024. 30266651

[183] Lambert CM, , HIF-1 inhibition decreases systemic vascular remodelling diseases by promoting apoptosis through a hexokinase 2-dependent mechanism, Cardiovasc Res. 88 (2010) 196–204, 10.1093/cvr/cvq152. 20498255

[184] Szwed A, , Regulation and metabolic functions of mTORC1 and mTORC2, Physiol. Rev 101 (2021) 1371–1426, 10.1152/physrev.00026.2020. 33599151 PMC8424549

[185] Gu M, , Inhibition of PIKfyve ameliorates the proliferation and migration of vascular smooth muscle cells and vascular intima hyperplasia by reducing mTORC1 activity, J. Cardiovasc Pharm 79 (2022) 739–748, 10.1097/fjc.0000000000001243. PMC9067083 35275098

[186] Sun X, , MTMR7 suppresses the phenotypic switching of vascular smooth muscle cell and vascular intimal hyperplasia after injury via regulating p62/mTORC1-mediated glucose metabolism, Atherosclerosis 390 (2024) 117470, 10.1016/j.atherosclerosis.2024.117470. 38342025

[187] Li Y, , DDX17 deficiency inhibits the proliferation and migration of vascular smooth muscle cells by inhibiting RHEB/mTORC1-mediated glycolysis and oxidative stress, J. Hypertens 43 (2025) 1232–1246, 10.1097/HJH.0000000000004043. 40439185

[188] Goncharov DA, , Mammalian target of rapamycin complex 2 (mTORC2) coordinates pulmonary artery smooth muscle cell metabolism, proliferation, and survival in pulmonary arterial hypertension, Circulation 129 (2014) 864–874, 10.1161/circulationaha.113.004581. 24270265 PMC3968690

[189] Zhu Y, , Platelet-Derived TGF (Transforming Growth Factor)-β1 enhances the aerobic glycolysis of pulmonary arterial smooth muscle cells by PKM2 (Pyruvate Kinase Muscle Isoform 2) upregulation, Hypertension 79 (2022) 932–945, 10.1161/hypertensionaha.121.18684. 35232222

[190] Kudryashova TV, , Profiling the role of mammalian target of rapamycin in the vascular smooth muscle metabolome in pulmonary arterial hypertension, Pulm. Circ 5 (2015) 667–680, 10.1086/683810. 26697174 PMC4671741

[191] Babicheva A, , mTOR signaling in pulmonary vascular disease: pathogenic role and therapeutic target, Int. J. Mol. Sci 22 (2021), 10.3390/ijms22042144. PMC7926633 33670032

[192] Sanches-Silva A, , Therapeutic potential of polyphenols in cardiovascular diseases: regulation of mTOR signaling pathway, Pharm. Res 152 (2020) 104626, 10.1016/j.phrs.2019.104626. 31904507

[193] Liu H, Chen YG, The interplay between TGF-β signaling and cell metabolism, Front. Cell Dev. Biol 10 (2022) 846723, 10.3389/fcell.2022.846723. 35359452 PMC8961331

[194] Calvier L, , PPARγ links BMP2 and TGFβ1 pathways in vascular smooth muscle cells, regulating cell proliferation and glucose metabolism, e1117, Cell Metab. 25 (2017) 1118–1134, 10.1016/j.cmet.2017.03.011. 28467929

[195] Yap C, , Six shades of vascular smooth muscle cells illuminated by KLF4 (Krüppel-Like Factor 4), Arterioscler. Thromb. Vasc. Biol 41 (2021) 2693–2707, 10.1161/atvbaha.121.316600. 34470477 PMC8545254

[196] Cao Y, , PFKFB3-mediated endothelial glycolysis promotes pulmonary hypertension, Proc. Natl. Acad. Sci. USA 116 (2019) 13394–13403, 10.1073/pnas.1821401116. 31213542 PMC6613097

[197] Haddad J, , In situ mapping of the glucose metabolism heterogeneity in atherosclerosis: correlation with 2-Deoxyglucose uptake, Mol. Imag 23 (2024) 15353508241280573, 10.1177/15353508241280573. PMC11577107 39568960

[198] Muhl L, , A single-cell transcriptomic inventory of murine smooth muscle cells, e2426, Dev. Cell 57 (2022) 2426–2443, 10.1016/j.devcel.2022.09.015. 36283392

[199] Zhang G, , Smooth muscle cell fate decisions decipher a high-resolution heterogeneity within atherosclerosis molecular subtypes, J. Transl. Med 20 (2022) 568, 10.1186/s12967-022-03795-9. 36474294 PMC9724432

[200] Ye W, , Single-cell RNA sequencing in human atherosclerotic plaques reveals a novel smooth muscle cell subtype that possesses multi differentiation potential and shapes the microenvironment, Clin. Exp. Med 25 (2025) 251, 10.1007/s10238-025-01735-7. 40668312 PMC12267379

[201] Mosquera JV, , Integrative single-cell meta-analysis reveals disease-relevant vascular cell states and markers in human atherosclerosis, Cell Rep. 42 (2023) 113380, 10.1016/j.celrep.2023.113380. 37950869 PMC12335892

